# Factors Associated With Thromboembolism in Neonates: A Systematic Review and Meta-Analysis

**DOI:** 10.1001/jamanetworkopen.2026.10908

**Published:** 2026-05-06

**Authors:** Marie-Claude Pelland-Marcotte, Norma Maria Pérez Herrera, Elisabeth Boileau, Heleen van Ommen, Rukhmi Bhat

**Affiliations:** 1 Department of Pediatrics, Centre Hospitalier Universitaire de Québec—Centre Mère-Enfant Soleil, Quebec City, Quebec, Canada; 2 Centre de Recherche du Centre Hospitalier Universitaire de Québec, Quebec City, Quebec, Canada; 3 Faculty of Medicine, Université Laval, Quebec City, Quebec, Canada; 4 Pediatric Hematology/Oncology, Erasmus MC Sophia Children’s Hospital, Rotterdam, the Netherlands; 5 Department of Pediatrics, Northwestern University Feinberg School of Medicine, Ann & Robert H. Lurie Children’s Hospital of Chicago, Chicago, Illinois; 6 Research Center of the CHU de Québec, Axe Reproduction, Santé de la Mère et de l’Enfant, Quebec City, Canada

## Abstract

**Importance:**

While venous thromboembolism (VTE) is increasingly recognized in neonates and prothrombotic risk factors are being identified, no risk assessment model currently exists to enable early identification of neonates at high risk of VTE. A thorough understanding of existing evidence is necessary to inform the development of a risk-assessment model.

**Objectives:**

To describe maternal, obstetrical, and neonatal risk factors associated with VTE in neonates.

**Data Sources:**

A literature search of Medline, Embase, CINAHL, and clinicaltrials.gov databases was performed on March 16, 2025, for peer-reviewed publications (1990-2025). Reference lists of included articles were screened to identify other potentially relevant publications.

**Study Selection:**

Peer-reviewed studies describing factors associated with venous thrombosis in neonates up to 44 weeks of corrected gestational age were included. A total of 282 reports were retrieved and assessed for eligibility.

**Data Extraction and Synthesis:**

Preferred Reporting Items for Systematic Reviews and Meta-Analyses and Meta-Analysis of Observational Studies in Epidemiology reporting guidelines were used to guide data abstraction. Studies were screened independently by 4 trained investigators. Data were pooled using a random-effects model.

**Main Outcomes and Measures:**

The primary outcome was the factors associated with venous thrombosis. Maternal and obstetrical and patient-related risk factors were considered; catheter-related risk factors are reported separately. Results are expressed using mean differences (MDs) or odds ratio (ORs) with 95% CIs. The degree of heterogeneity (ie, τ^2^) was estimated using the restricted maximum-likelihood model estimator. Risk of bias in included studies was assessed using the Risk of Bias in Non-randomised Studies of Interventions tool.

**Results:**

A total of 60 studies (3 366 507 neonates) were included; most were retrospective cohort studies (26 studies [43%]) or case-control studies (15 studies [25%]). The following risk factors were significantly associated with venous thrombosis: preeclampsia (OR, 2.66; 95% CI, 1.70-4.16; *P* < .001; τ^2^ = 0.00), low birth weight (OR, 2.05; 95% CI, 1.41-3.00; *P* < .001; τ^2^ = 0.00), cardiac disease (OR, 5.90; 95% CI, 1.16-30.05; *P* = .03; τ^2^ = 1.27), and infection (OR, 2.90; 95% CI, 1.88-4.48; *P* < .001; τ^2^ = 0.33). Several other factors were identified in multiple studies, but with inconsistent results, including maternal diabetes, gestational age and prematurity, asphyxia, mechanical ventilation, surgery, and elevated hemoglobin or hematocrit levels.

**Conclusions and Relevance:**

In this systematic review and meta-analysis, preeclampsia, low birth weight, cardiac disease, and infection were identified as risk factors of neonatal venous thrombosis. Heterogeneity between studies, lack of thrombosis outcome definition, and limited studies addressing common conditions and medications were identified, highlighting the need for further studies and risk stratification.

## Key Points

**Question:**

What are the factors associated with neonatal venous thrombosis?

**Findings:**

In this systematic review and meta-analysis of 60 studies including 3 366 507 neonates, preeclampsia, low birth weight, neonatal infection, and cardiac disease were associated with venous thrombosis in neonates. Several putative factors were analyzed in fewer than 3 studies, and their impact could not be explored.

**Meaning:**

These findings suggest that preeclampsia, low birth weight, cardiac disease, and infection may be risk factors for neonatal venous thrombosis, although limited data regarding common conditions and medications persist, highlighting the need for further studies and risk stratification.

## Teaser

This systematic review and meta-analysis evaluates maternal, obstetrical, and neonatal risk factors associated with venous thromboembolism in neonates.

## Introduction

The incidence of venous thromboembolism (VTE) is rising in children.^1,2^ Neonates are particularly at risk of thrombosis within the pediatric population.^3^ VTE may lead to serious complications, including organ dysfunction (eg, systemic hypertension and kidney dysfunction following kidney thrombosis), portal hypertension, postthrombotic syndrome, and even death,^4,5,6^ while its treatment may lead to systemic and/or intracranial bleeding, especially in premature infants.^7^

Several prospective and retrospective studies have explored the epidemiology of neonatal VTE. Reported incidences varying widely, between 0.2% to 75%,^2,8,9,10,11^ depending on the population studied, whether clinically unsuspected thromboses are considered, and if active radiological screening is performed. A pooled analysis of 82 studies^12^ reported a 10.6% cumulative incidence of VTE in neonates hospitalized in neonatal intensive care units (NICU), highlighting the high burden of VTE in this population. Understanding factors associated with VTE in this unique neonatal population is important because it may help identify subpopulations at particularly high risk who could benefit from preventative measures, such as strategies for optimal use of central venous catheters (CVC).

Thus, we performed a systematic review and meta-analysis of the literature to synthesize the factors associated with VTE in neonates, incorporating data from prospective and retrospective cohort studies, case-control studies, and cross-sectional studies. We did not state predefined hypothesis prior to engaging with the literature.

## Methods

The systematic review and meta-analysis was reported in accordance with the Preferred Reporting Items for Systematic Reviews and Meta-Analyses (PRISMA) and the Meta-Analysis of Observational Studies in Epidemiology (MOOSE) reporting guidelines.^13^ The protocol was recorded on the PROSPERO registry (CRD42024518801).^14^ This systematic review benefited from a waiver from research ethics board approval due to institutional policies for systematic review.

### Data Sources and Search

A professional librarian performed a search, not limited by language, from 1990 to March 16, 2025, in the Medline, Embase, CINAHL, and clinicaltrials.gov databases. We considered articles after 1990 only to reflect contemporary neonatal care characterized by increased survival of premature and critically ill infants, notably following integration of surfactant replacement therapy and increased expertise in cardiac surgeries for congenital heart disease.^15,16^ The reference lists of included articles were screened to identify other potentially relevant publications. eTable 1 in Supplement 1 shows the databases search strategy.

### Study Selection

We included nonrandomized trials, retrospective and prospective cohort studies, case-control studies and case series (≥10 patients) published in peer-reviewed journals. Secondary analysis of randomized clinical trials, or randomized controlled trials in which VTE was a primary or secondary outcome, were also eligible. Studies were selected if at least 90% of the included population were neonates, defined as full-term infants 28 days of age or older or preterm infants with corrected gestational age of 44 weeks or less, and if the studies reported maternal, obstetrical, or neonatal factors associated with VTE, including both symptomatic and clinically unsuspected events. CVC-related factors will be reported separately. We did not limit our search to English-language publications and used software-based translation for articles in languages other than English, French, Spanish, or Dutch.

Titles and abstracts of studies retrieved by the search strategy, and full text of potentially eligible studies were screened independently by 2 of 4 trained investigators (M.C.P.M., N.P., E.B., and R.B.), using the Covidence systematic review software (Veritas Health Innovation). Any disagreement over study eligibility was resolved through discussion, leading to a consensus.

### Data Extraction and Quality Assessment

Data extraction was conducted independently by 2 of 4 investigators (M.C.P.M., N.P., E.B., and R.B.) using a standardized Microsoft Excel version 2603 form (Microsoft Corporation). Extracted information included study setting, design, sample size, patient characteristics, VTE outcome definition, use of surveillance imaging, and factors associated with VTE. We applied the following strategy during data extraction: (1) extraction of factors assessed in multivariable analysis (felt to be at lesser risk of confounding); (2) extraction of factors evaluated as the main objective of the study (felt to be at less risk of selective reporting); and (3) systematic extraction of age, birth weight, and gestational age when available (to allow stratified analyses). This strategy, while excluding variables considered in univariate analysis alone, was pursued to minimize the risk of type I error. For each factor, we extracted the variable definition and, when applicable, measurement time points and laboratory assays used. For risk factors related to the use of medication or blood products, we extracted the timing and dose of administration. The corresponding authors of the studies were contacted via email up to 3 times for important missing data for study eligibility or data abstraction.

Two members of the review team (M.C.P.M, N.P., E.B., and R.B.) independently evaluated the risk of bias using the Risk of Bias in Non-randomised Studies of Interventions (ROBINS-I) tool.^17,18^ The tool was adapted so that risk of bias was classified as high, low, or unclear (rather than low, moderate, high, or serious) for each of the following domains: confounding, selection of participants, classification of exposure and risk factors, deviation from intended procedures, missing data, measurement of outcomes, and selective reporting. As suggested by the tool’s authors, confounders of interest were determined a priori and included gestational age and prematurity, severe medical conditions (cardiac disease, sepsis, and mechanical ventilation), or use of a global score of neonatal disease severity such as the Score for Neonatal Acute Physiology (SNAP-II),^19^ and presence of a CVC.

### Statistical Analysis

Data were presented descriptively, using means and SDs for continuous data, and proportions for categorical data, as appropriate. The impact of risk factors described in 3 or more studies was evaluated with random-effects meta-analysis; results are expressed using a mean difference (MD) or odds ratio (OR) with 95% CIs and displayed using Forest plots. The degree of heterogeneity (ie, τ^2^) was estimated using the restricted maximum-likelihood estimator.^20^ The certainty of evidence was assessed using the GRADE (Grading of Recommendations, Assessment, Development, and Evaluation) approach, with VTE as the sole outcome of interest.^21,22^

We planned to conduct the following subgroup analyses: (1) factors associated with VTE in premature neonates; (2) factors associated with VTE in neonates with cardiac disease; and (3) factors associated with symptomatic VTE (without considering clinically unsuspected VTE) because the clinical relevance of clinically unsuspected VTE is unclear.^23,24^ We hypothesized that factors associated with VTE would vary in neonates with prematurity and cardiac disease, due to the evolving hemostatic system and distinct catheter-related and treatment exposures, respectively.^25^ We also performed a sensitivity analysis using only factors reported in prospective studies to test the robustness of our findings because we suspected that factors identified in retrospective studies were more vulnerable to selective reporting.

All analyses were performed using the Review Manager (RevMan) computer program version 5.4 (The Cochrane Collaboration) and Jamovi version 2.6 (open source). A 2-sided *P* value less than .05 was considered statistically significant.

## Results

The search strategy produced 13 370 unique references, of which 282 full-text articles were retrieved (eFigure 1 in Supplement 1). Sixty studies (totaling 3 366 507 neonates) were included.^10,11,26,27,28,29,30,31,32,33,34,35,36,37,38,39,40,41,42,43,44,45,46,47,48,49,50,51,52,53,54,55,56,57,58,59,60,61,62,63,64,65,66,67,68,69,70,71,72,73,74,75,76,77,78,79,80,81,82,83^ eTable 2 and eTable 3 in Supplement 1 describe the characteristics of included studies and give a study-level summary of included studies, respectively. Most studies were retrospective (26 studies^27,28,33,34,38,39,41,42,43,44,48,52,53,54,55,56,57,58,59,60,68,69,72,73,75,81^ [43%]) or case-control studies (15 studies^10,11,29,30,31,32,36,45,49,71,74,76,78,79,83^ [25%]), and 23 studies^10,11,26,31,33,36,37,38,40,41,44,46,53,55,58,59,63,70,71,73,81,82,83^ (38%) were published within the last 5 years. While VTE definition often did not differentiate symptomatic and clinically unsuspected VTE, 29 studies^35,40,41,42,43,45,46,47,48,49,50,52,54,57,58,61,62,63,64,65,66,67,68,70,74,77,79,81,82^ (48%) performed at least 1 surveillance imaging for VTE screening.

The risk of bias is presented in eTable 4 in Supplement 1. Most studies (52 studies^10,11,26,27,28,29,30,31,32,33,34,35,36,37,38,39,40,41,43,44,45,46,47,48,49,50,52,53,54,55,56,57,58,59,60,61,62,63,64,65,66,67,68,69,70,71,74,75,76,78,82,83^ [87%]) had serious risk of bias in at least 1 domain, frequently risk of confounding (serious risk: studies 40 studies^11,26,27,28,31,32,33,34,35,36,37,39,40,41,43,44,45,48,49,50,52,53,54,55,56,58,59,60,61,62,63,64,65,69,70,71,74,75,76,78,82,83^ [66%]; unclear risk: 1 study^37^ [2%]).

### Maternal and Obstetrical Risk Factors

**Figure 1.**
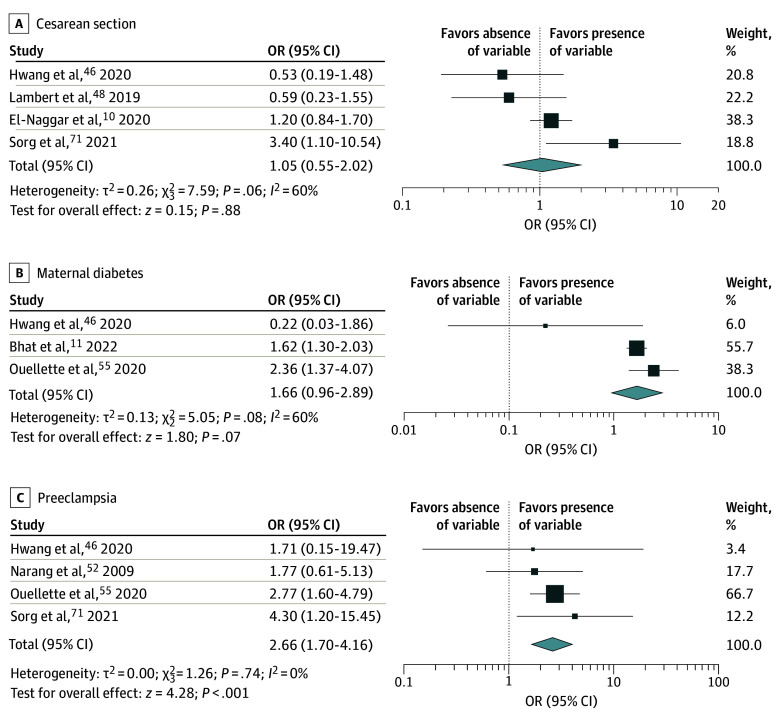
Forest Plot of the Association of Maternal and Obstetrical Variables With the Risk of Venous Thrombosis Analyses were performed with study-level multivariate regression analyses (adjusted odds ratios [ORs] and 95% CIs).

Preeclampsia was associated with neonatal venous thrombosis (OR, 2.66, 95% CI, 1.70-4.16; *P* < .001; τ^2^ = 0.00; 4 studies^46,55,52,71^), while maternal diabetes (OR, 1.66; 95% CI, 0.96-2.89; τ^2^ = 0.13; *P* = .07; 3 studies^11,46,55^) and cesarean section (vs vaginal delivery; OR, 1.05; 95% CI, 0.55-2.02; *P* = .88; τ^2^ = 0.26; 4 studies^10,46,48,71^) were not statistically associated with thrombosis (Figure 1). The GRADE summary of findings for all quantitative analyses is displayed in Table 1.

**Table 1.** GRADE Summary of Findings

Study population	No. of studies (No. of participants)^a^	Risk of bias	Inconsistency	Indirectness	Imprecision	OR (95% CI)	Certainty of evidence
Cesarean section	4^10,46,48,71^ (40 925)	Very serious^b^	Very serious	Not serious	Serious	1.05 (0.55 to 2.20)	Very low^c^
Maternal diabetes	3^11,46,55^ (3 106 857)	Very serious^d^	Very serious	Not serious	Serious	1.66 (0.96 to 2.89)	Very low^c^
Preeclampsia	4^46,52,55,71^ (3 102 008)	Very serious^b^	Not serious	Not serious	Serious	2.66 (1.70 to 4.16)	Very low^c^
Low birth weight	4^34,43,76,78^ (1928)	Very serious^e^	Not serious	Not serious	Not serious	2.05 (1.41 to 3.00)	Very low^c^
Birth weight (in g)	11^37,41,46,50,52,55,61^ ^68,79,81,83^ (3 103 368)	Serious^f^	Not serious	Not serious	Serious	Mean reduction, 43.9 g (95% CI, −79.9 to −7.9 g)	Very low^c^
Gestational age							
Prematurity vs born at term	6^37,40,46,48,52,76^(2436)	Very serious^e^	Serious	Not serious	Serious	1.03 (0.87 to 1.22)	Very low^c^
Weeks of gestation	5^46,50,52,61,83^ (701)	Very serious^e^	Very serious	Not serious	Serious	Mean difference, −0.45 wk (95% CI, −1.71 to 0.82 wk)	Very low^c^
Assigned sex at birth	20^26,37,38,40,46,48,49,50,52,54,55,61,65,68,76,77,78,79,82,83^ (3 307 240)	Very serious^g^	Serious	Not serious	Not serious	1.17 (0.94 to 1.45) for males vs females	Very low^c^
Asphyxia or maladaptation to extrauterine life	4^36,49,71,76^ (1254)	Very serious^e^	Serious	Not serious	Very serious	5.88 (0.85 to 40.72)	Very low^c^
Cardiac disease	3^48,55,83^ (3 102 439)	Very serious^b^	Serious	Not serious	Very serious	5.90 (1.15 to 30.05)	Very low^c^
Infection	10^10,11,26,29,32,33,36^ ^38,40,55^ (3 349 324)	Very serious^g^	Very serious	Not serious	Not serious	2.90 (1.88 to 4.48)	Very low^c^
Mechanical ventilation	4^10,11,29,38^ (246 228)	Very serious^e^	Not serious	Not serious	Serious	2.00 (0.52 to 7.60)	Very low^c^
Surgery	4^11,29,44,63^ (10 194)	Very serious^b^	Serious	Not serious	Very serious	3.05 (0.88 to 10.51)	Very low^c^
Elevated hemoglobin or hematocrit	3^46,49,52^ (394)	Very serious^d^	Serious	Not serious	Serious	1.52 (0.63 to 3.68)	Very low^c^
Platelet count	3^40,46,52^ (481)	Very serious^b^	Not serious	Not serious	Very serious	0.99 (0.99 to 1.00)	Very low^c^

Abbreviations: GRADE, Grading of Recommendations, Assessment, Development, and Evaluation; OR, odds ratio.

^a^ All studies were nonrandomized studies.

^b^ Most studies were at serious risk of bias (bias due to confounding and bias in outcome measurement and selective reporting).

^c^ Downgraded for inconsistency and/or imprecision.

^d^ Most studies were at serious risk of bias (bias due to confounding and bias in outcome measurement).

^e^ Most studies were at serious risk of bias (bias due to confounding, selection bias, and bias in classification of exposure and in outcome measurement).

^f^ Some studies were at serious risk of bias (bias due to confounding, outcome measurement, and selective reporting).

^g^ Most studies were at serious risk of bias (bias due to confounding, selection bias, bias in classification of exposure and in outcome measurement, and risk of selective reporting).

### Demographic Variables

Among neonates hospitalized to the NICU, low birth weight (LBW) was associated with VTE, both in studies where birth weight was reported as a categorical variable (low vs normal birth weight: OR, 2.05; 95% CI, 1.41 to 3.00; *P* < .001; τ^2^ = 0.00; 4 studies^34,43,76,78^) or as a continuous variable (MD, −43.91 g; 95% CI, −79.94 to −7.87 g; *P* = .02; τ^2^ = 0.00; 11 studies^37,41,46,50,52,55,61,68,79,81,83^).

Conversely, gestational age, described continuously (MD in weeks of gestational age: −0.45 weeks; 95%CI, −1.71 to 0.82 weeks; *P* = .49, τ^2^ = 1.49; 5 studies^46,50,52,61,83^) or categorized between prematurity and born at term (OR, 1.03; 95% CI, 0.87 to 1.22; *P* = .74; τ^2^ = 0.03; 6 studies^37,40,46,48,52,76^) and sex (male vs female: OR, 1.15; 95% CI, 0.97 to 1.36; *P* = .11; τ^2^ = 0.02; 20 studies^26,37,38,40,46,48,49,50,52,54,55,61,65,68,76,77,78,79,82,83^) were not significantly associated with VTE (eFigures 2-4 in Supplement 1).

### Neonatal Factors

**Figure 2.**
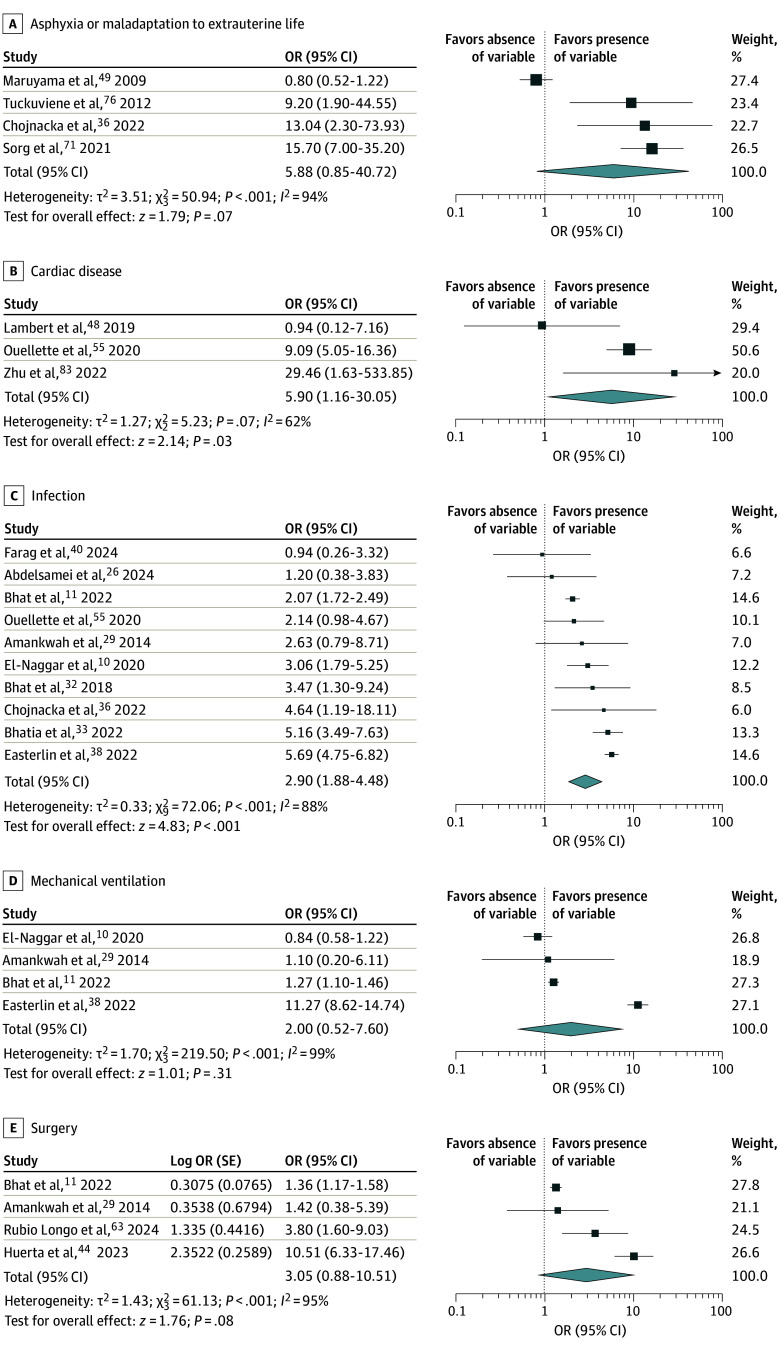
Forest Plot of the Association of Postnatal Conditions With the Risk of Venous Thrombosis Analyses were performed with study-level multivariate regression analyses (adjusted odds ratios [ORs] and 95% CIs).

Cardiac disease (OR, 5.90, 95% CI, 1.16-30.05; *P* = .03, τ^2^ = 1.27; 3 studies^48,55,83^) and infection (OR, 2.90; 95% CI, 1.88-4.48; *P* < .001; τ^2^ = 0.33; 10 studies^10,11,26,29,32,33,36,38,40,55^) were significantly associated with VTE (Figure 2). Asphyxia (OR, 5.88; 95% CI, 0.85-40.72; *P* = .07; τ^2^ = 3.51; 4 studies^36,49,71,76^), mechanical ventilation (OR, 2.00; 95% CI, 0.52-7.60; *P* = .31; τ^2^ = 1.70; 4 studies^10,11,29,38^), and surgery (OR, 3.05; 95% CI, 0.88-10.51; *P* = .08; τ^2^ = 1.43; 4 studies^11,29,44,63^) were more frequent in neonates with VTE, but did not reach statistical significance (Figure 2). The association of respiratory distress syndrome, necrotizing enterocolitis, and cholestasis with VTE development was reported in fewer than 3 studies and was not analyzed. Additional data reported in fewer than 3 studies are shown in eTable 5 in Supplement 1.

### Laboratory Parameters

Nine studies (1810 neonates)^26,30,45,46,49,50,52,77,84^ explored the association of laboratory values with the risk of VTE and are summarized in Table 2. Increased hemoglobin and hematocrit, reported continuously (in g/dL)^49^ or dichotomized at hematocrit greater than 55%^52^ or greater than 60%,^46^ was not significantly associated with VTE (OR, 1.52; 95% CI, 0.63-3.68; *P* = .13; τ^2^ = 0.32; 3 studies^46,49,52^). The association of platelet count (per 10^3^ μL) was evaluated in 4 studies,^40,46,50,52^ of which 3 studies^40,46,52^ could be synthesized and showed no significant association with venous thrombosis (OR, 0.99; 95% CI, 0.99-1.00; *P* = .09; τ^2^ = 0.00). Other laboratory parameters, such as blood group, C-reactive protein, electrolytes, and kidney function tests, and various thrombophilic traits, were evaluated in fewer than 3 studies and were not analyzed.

**Table 2.** Laboratory Markers Associated With Neonatal Venous Thrombosis

Reference	Study design	Population (sample size, No.)	Definition, laboratory assay, units	Time point for testing	Values in patients with VTE vs controls, mean (SD)	*P* value	Proportion of patients with abnormal values in patients with VTE vs controls, No./total No.	OR (95% CI)	*P* value
Blood group									
Hwang et al,^46^ 2020	PCS	General (137)	Type O vs non–type O	NR	NR	NA	7/26 vs 30/111	0.90 (0.33-2.49)	.84
C-reactive protein									
Abdelsamei et al,^26^ 2024	PCS	General (886)	Elevation of C-reactive protein in mg/L	NR	NR	NA	33/36 vs 304/850	1.02 (1.01-1.03)	.001
Cholette et al,^84^ 2007	PCS	Neonates undergoing cardiac surgery (22)	Wide-range C-reactive protein >15 vs <15 mg/L	Preoperative	NR	NA	3/4 vs 2/18	NR	.02
Electrolytes and kidney function									
Hwang et al,^46^ 2020	PCS	General (137)	Sodium >145 mEq/L	NR	NR	NA	6/26 vs 22/111	0.96 (0.31-2.98)	.95
Hwang et al,^46^ 2020	PCS	General (137)	Calcium >10 mg/dL	NR	NR	NA	6/26 vs 9/111	4.39 (1.26-15.32)	.02
Hwang et al,^46^ 2020	PCS	General (137)	BUN to creatinine ratio >20:1	NR	NR	NA	16/26 vs 65/111	0.97 (0.36-2.60)	.95
Maruyama et al,^49^ 2012	CCS	Prematurity or low birth weight (47)	Excess base, in mmol/L	Birth	−7.5 (4.1) vs −9.3 (4.1) mmol/L	.19	NR	3.95 (1.41-14.39)	.008
Hematocrit and hemoglobin									
Hwang et al,^46^ 2020	PCS	General (137)	Hematocrit >60%	NR	NR	NA	1/26 vs 5/111	1.07 (0.10-11.11)	>.99
Maruyama et al,^49^ 2012	CCS	Prematurity or low birth weight (47)	Hemoglobin in g/dL	Day 3 of life	12.6 (1.3) vs 11.4 (1.5) g/dL	.02	NR	1.05 (0.79-1.38)	.74
Narang et al,^52^ 2009	RCS	Prematurity or low birth weight (210)	Hematocrit >55%	Within first wk of life	NR	NA	NR	3.66 (1.13-11.79)	.03
Platelet count									
Hwang et al,^46^ 2020	PCS	General (137)	Thrombocytopenia <100 000/μL	NR	NR	NA	1/26 vs 6/111	1.17 (0.11-12.81)	>.99
Mehta et al,^50^ 1992	RCS	General (42)	Platelet count in 1000/mm^3^	At catheter placement	184 (81.7) vs 285 (202) 1000/mm^3^	NR	NR	NR	.15
Narang et al,^52^ 2009	RCS	Very low birth weight infants requiring central venous catheters (210)	Highest platelet count per 10^3^ μL	Within first wk of life	193 (57) vs 238 (70) per 10^3^ μL	.005	NR	0.94 (0.86-1.03)	>.05
Farag et al,^40^ 2024	PCS	General (134)	NR	NR	152.0 (200.8) vs 220.1 (89.7)	NR	NR	0.995 (0.989-1.00)	.07
Thrombophilias and coagulation proteins									
Aronis et al,^30^ 2002	CCS	Full-term infants with CNS thrombosis (106)	Any of FVL, PGM, or MTHFR	NR	NR	NA	3/13 vs 4/38	2.55 (0.49-13.33)	NR
Aronis et al,^30^ 2002	CCS	Full-term infants with CNS thrombosis (106)	PGM	NR	NR	NA	1/13 vs 1/38^a^	2.47 (0.28-21.76)	NR
Hundsdoerfer et al,^45^ 2003	PCS	General (307)	Homozygous or compound heterozygous FVL and FII G20210A vs heterozygous, by PCR	At birth	NR	NA	4/129 vs 0/178	NR	.02
Maruyama et al,^49^ 2012	CCS	Prematurity or low birth weight (47)	Fibrinogen, in mg/dL	At birth	130 (93) vs 194 (55) mg/dL	NR	NR	0.61 (0.28-1.12)	.12
Mehta et al,^50^ 1992	PCS	General (42)	Antithrombin levels, by immunologic quantitation, in U/mL	At catheter placement	0.32 (0.08) vs 0.60 (0.31) U/mL	NR	NR	NR	.001
Turebylu et al,^77^ 2007	PCS	General (53)	FVL, PGM, MTHFR 667 or 1298	NR	NR	NA	16/17 vs 47/55	NR	>.05

Abbreviations: BUN, blood urea nitrogen; CCS, case-control study; CNS, central nervous system; FVL, factor V Leiden; MTHFR, methyl tetra hydro folate reductase; NA, not applicable; NR, not reported; OR, odds ratio; PCR, polymerase chain reaction; PCS, prospective cohort study; PGM, prothrombin G20210A; RCS, retrospective cohort study; VTE, venous thromboembolism.

^a^ OR, 2.47 (95% CI, 0.28-21.76).

### Medication and Blood Products

Overall, 9 studies (202 810 neonates)^38,40,48,49,53,57,60,62,82^ investigated the association of total parenteral nutrition, postnatal medication (steroids or recombinant factor VIIa) or blood product administration in neonates (any, packed red blood cells, fresh frozen plasma, activated 4-factor prothrombin complex concentrates, and antithrombin concentrates) with VTE, with no intervention examined in at least 3 studies. Therefore, results are summarized descriptively in Table 3.

**Table 3.** Medications or Blood Products Associated With Neonatal VTE

Reference	Study design	Population (sample size, No.)	Definition of VTE	Medication or blood products	Proportion of patients with abnormal values in patients with VTE vs Controls	OR (95% CI)	*P* value
Total parenteral nutrition							
Easterlin et al,^38^ 2022	RCS	General (201 033)	Based on *ICD-10* discharge codes, all VTE sites	Total parenteral nutrition	2437/2720 vs 74 845/198 313	17.46 (13.40-22.67)	NR
Xiong et al,^82^ 2025	PCS	Surgery for congenital anomaly (188)	Occlusive or nonocclusive thrombus identified by Doppler ultrasonography	Total parenteral nutrition	Median (IQR) duration, 16 (11-34) vs 12 (7-20) d	2.09 (0.44-9.95)	.36
Blood products							
Farag et al,^40^ 2024	PCS	General (142)	Echodense structure seen by ultrasonography within heart of vessels surrounding CVC	Transfusion of packed red blood cells	15/17 vs 46/125	5.77 (1.01-32.84)	.048
Lambert et al,^48^ 2019	RCS	General (766)	Clinically identifiable VTE	Any blood product	NR^a^	1.48 (0.57-3.88)	NR
Maruyama et al,^49^ 2012	CCS	Extremely low birth weight infants (47)	Venous occlusion diagnosed by ultrasonography	Fresh frozen plasma (>50 mL/kg by day 5)	9/13 vs 12/34	5.88 (1.12-41.81)	.04
Navaratnam et al,^53^ 2023	RCS	Undergoing cardiac surgery with cardiopulmonary bypass (86)	Radiologically proven VTE	Activated 4-products prothrombin concentrates	7/43 vs 4/43	1.88 (NR)	.88
Petäjä et al,^57^ 1999	RCS	Undergoing cardiac surgery (242)	Radiologically proven VTE (phlebography or echocardiograms)	Antithrombin supplementation	2/82 vs 101/160	NR	.20
Medications							
Puetz et al,^60^ 2009	RCS	Critically ill neonates (234)	NR	Activated factor VIIa	10/134 vs 7/100	1.00 (NR)	NR
Röhr et al,^62^ 2014	PCS	Low birth weight infants (72)	Venous occlusion diagnosed by ultrasonography	Steroids	8/34 vs 1/38	11.4 (1.3-96.6)	.01
Xiong et al,^82^ 2025	PCS	Surgery for congenital anomaly (188)	Occlusive or nonocclusive thrombus identified by Doppler ultrasonography	Any vasoactive drug	44/135 vs 9/53	2.64 (0.94-7.39)	.07

Abbreviations: CCS, case-control; CVC, central venous catheter; *ICD-10*, *International Statistical Classification of Diseases and Related Health Problems, Tenth Revision*; NR, not reported; PCS, prospective cohort study; OR, odds ratio; RCS, retrospective cohort study; VTE, venous thromboembolism.

^a^ OR, 1.48 (95% CI, 0.57-3.88).

### Subgroup and Sensitivity Analyses

Data were insufficient to perform subgroup analyses because no factor was evaluated in at least 3 studies for each of the selected subpopulations (prematurity and cardiac disease). Similarly, we could not perform analyses for symptomatic thromboses because several reports (17 studies^33,39,49,50,52,53,58,62,64,67,73,74,76,77,78,80,81^ [28%]) did not report the definition of VTE, and an additional 34 studies^10,11,29,30,31,32,34,35,36,37,38,40,42,43,44,45,46,48,51,54,57,59,60,61,63,65,66,68,69,71,72,75,79,82^ (57%) did not report symptomatic and clinically unsuspected VTEs separately. In the sensitivity analysis comprising only prospective studies, the association of gestational age (3 studies^46,50,61^), sex (9 studies^26,37,40,46,50,61,65,77,82^), and birth weight (4 studies^37,46,50,61^) with VTE could be summarized; none were associated with VTE (eFigure 5 in Supplement 1).

## Discussion

To our knowledge, this meta-analysis of factors associated with neonatal VTE is the largest to date. The published literature has identified several putative maternal and obstetrical, demographic, neonatal, and postnatal risk factors for neonatal VTE; however, replication of results across different studies has proved challenging. Pooled results from multiple studies suggest that preeclampsia, LBW, cardiac disease, and infection are associated with neonatal VTE.

It is biologically plausible that preeclampsia and LBW are associated with VTE. Preeclampsia, characterized by abnormal placental vasculature, leads to hypoperfusion and hypoxia of the feto-placental unit and results in blood-sparing, where blood is diverted to the brain and heart, accompanied by vasoconstriction in other organs and limbs. It may lead to intrauterine growth restriction, which is associated with several neonatal adverse outcomes such as sepsis, respiratory distress syndrome, and polycythemia. Similarly, the association of cardiac disease with thrombosis is widely recognized in older children.^85^ Almost 50% of infants younger than 6 months and 30% of older children with thromboembolic disease have underlying cardiac disorders.^86,87^ Hypothesized mechanisms include alterations to blood flow and venous stasis, disturbances of the vascular endothelium related to surgery, intravascular sutures, or vascular manipulation. Finally, growing evidence highlights extensive crosstalk between sepsis and coagulopathy. In response to endotoxins and other pathogen-associated signals, tissue factor expression is upregulated in monocytes and endothelial cells, resulting in increased secretion of proinflammatory cytokines and increased thrombin generation. In turn, thrombin further upregulates these processes through platelet activation, stimulation of procoagulant and anticoagulant proteins, and inducing proinflammatory cytokines, anti-inflammatory cytokines, and mitogenic responses. Moreover, sepsis is associated with depressed levels of natural anticoagulants such as antithrombin and inhibition of fibrinolysis.^88,89,90^

In addition to these variables, it is likely that several other clinical characteristics impact the risk of VTE. This review highlighted significant clinical and methodological heterogeneity that may have resulted in discrepant results. For example, surgery, a well-established prothrombotic risk factor in older children^91,92^ and adults,^93^ was not significantly associated with neonatal VTE, due to negative findings in 1 study^29^ and large confidence intervals in other reports. Similarly, asphyxia and maladaptation to extrauterine life were significantly associated with VTE in 3 of 4 studies^36,71,76^ but had no impact in 1 study.^49^ It is possible that these differences are attributable to inconsistent variable definition (for example, surgery type or definition of asphyxia), differences in study population, and study size, which may have lacked statistical power to detect these associations.

Strikingly, for several variables, data were insufficient to perform meta-analyses. For example, meta-analyses were not possible for respiratory distress syndrome, necrotizing enterocolitis, or cholestasis, despite these being common conditions in the NICU. This limitation has important implications not only for the identification of neonates at high risk of VTE, but also for informed clinical decision-making. Notably, individual studies in LBW infants showed an increased risk of thrombosis following exposure to fresh frozen plasma or steroids. If validated, these findings could inform clinical practice by guiding the risks and benefits of these interventions or, at a minimum, raising awareness of the risk of iatrogenic VTE.

A recent meta-analysis reported a cumulative incidence of VTE of 10.6% in neonates admitted to the NICU,^12^ echoing other reports that highlight the increasing burden of thrombotic disease in neonates and children.^94^ Most factors identified in our meta-analysis are likely nonmodifiable; however, their recognition early in the neonates’ clinical course may help clinicians stratify risk and implement targeted prevention strategies. Future research should focus on identifying reliable biomarkers of thrombosis and on evaluating the impact of common NICU interventions on thrombosis risk. As understanding of the pathophysiology and epidemiology of neonatal VTE continues to evolve, efforts should shift toward the development of risk-assessment models, perhaps incorporating machine learning approaches applied to large datasets. Such models may enable earlier identification of neonates at high risk of VTE earlier in their clinical course and support the evaluation of preventable strategies in this vulnerable group.

### Strengths and Limitations

The strengths of our systematic review include a thorough search from 4 distinct databases, rigorous methodology prepublished in an open registry for optimal transparency, and strict adherence to reporting guidelines. However, a few limitations from this systematic review merit discussion. First, studies included were retrospective in design, limited by small sample sizes and/or had a serious risk of bias, thereby limiting the strength and certainty of our conclusions. Additionally, there is a potential for risk of reverse causality because several studies were unable to precisely determine the timing of different variables (eg, infection and thrombosis) in relation to the thrombotic event. We mitigated that risk by performing sensitivity analysis including prospective studies alone. However, we were unable to perform a sensitivity analysis by risk of bias as very few studies met the assessment criteria. Further prospective research, with a priori variable definitions and comprehensive data collection, is necessary to confirm our findings and clarify the association of neonatal thrombosis with other measured and unmeasured variables. Second, our review could not account for the heterogeneity of neonatal care because most studies focused on the general NICU population without stratifying by comorbidity. This limited our ability to identify factors from specific subpopulations, where effects may vary by settings, for example using stratified analyses. Third, there is a risk of publication bias or, more importantly, selective reporting, if only statistically significant factors are reported while omitting other clinical characteristics not associated with VTE. We carefully selected our data extraction strategy to reduce this risk while selecting factors evaluated in adjusted analyses or a priori established as primary or secondary study outcomes. However, given the substantial number of factors evaluated, it is plausible that publication bias affected our results.

## Conclusions

In this systematic review and meta-analysis of factors associated with venous thrombosis in neonates, we identified several risk factors for neonatal VTE, including preeclampsia, LBW, infection, and cardiac disease. However, important discrepancies existed between studies on clinically relevant variables such as gestational age, surgery, asphyxia, and mechanical ventilation. Comprehensive prospective research is urgently needed to improve risk prediction and develop prevention strategies for VTE in neonates.

## Notes

Supplement 1. eTable 1. Search strategy used in databases eFigure 1. Flow chart of included studies eTable 2. Characteristics of included studies eTable 3. Study-level summary of included studies eTable 4. Risk of bias in included studies eFigure 2. Forest plot of the effect of birth weight on the risk of venous thrombosis eFigure 3. Forest plot of the effect of gestational age on the risk of venous thrombosis eFigure 4. Forest plot of the effect of sex (male vs. female) on the risk of venous thrombosis eTable 5. Additional clinical factors associated with neonatal venous thrombosis eFigure 5. Sensitivity analyses of the association between VTE and demographic variables—prospective studies only eReferences

Supplement 2. Data Sharing Statement
