# Supplementary material for: Factors Associated With Thromboembolism in Neonates: A Systematic Review and Meta-Analysis
Source: JAMA Netw Open. 2026 May 6;9(5):e2610908. doi: 10.1001/jamanetworkopen.2026.10908 (PMC13150647; doi:10.1001/jamanetworkopen.2026.10908)
Supplement: Supplement 1. — eTable 1. Search strategy used in databases eFigure 1. Flow chart of included studies eTable 2. Characteristics of included studies eTable 3. Study-level summary of included studies eTable 4. Risk of bias in included studies eFigure 2. Forest plot of the effect of birth weight on the risk of venous thrombosis eFigure 3. Forest plot of the effect of gestational age on the risk of venous thrombosis eFigure 4. Forest plot of the effect of sex (male vs. female) on the risk of venous thrombosis eTable 5. Additional clinical factors associated with neonatal venous thrombosis eFigure 5. Sensitivity analyses of the association between VTE and demographic variables—prospective studies only eReferences [file jamanetwopen-e2610908-s001.pdf]

Supplemental Online Content

Pelland-Marcotte MC, Pérez Herrera NM, Boileau E, van Ommen H, Bhat R. Factors associated with venous thromboembolism in neonates : a systematic review and meta-analysis. *JAMA Netw. Open*. 2026 ;9(5) :e2610908. doi:10.1001/jamanetworkopen.2026.10908

eTable 1. Search strategy used in databases

eFigure 1. Flow chart of included studies

eTable 2. Characteristics of included studies

eTable 3. Study-level summary of included studies

eTable 4. Risk of bias in included studies

eFigure 2. Forest plot of the effect of birthweight on the risk of venous thrombosis

eFigure 3. Forest plot of the effect of gestational age on the risk of venous thrombosis

eFigure 4. Forest plot of the effect of sex (male vs. female) on the risk of venous thrombosis

eTable 5. Additional clinical factors associated with neonatal venous thrombosis

eFigure 5. Sensitivity analyses of the association between VTE and demographic variables – prospective studies only

eReferences

This supplemental material has been provided by the authors to give readers additional information about their work.

© 2026 Pelland-Marcotte MC et al. *JAMA Network Open*.

eTable 1. Search strategies used in databases

a) MEDLINE

Concepts	Research strategy keywords	# Research
**Neonates**		
Controlled vocabulary	exp infant/ or intensive care units, neonatal/	1
Free text	(neonate* or neonatal or infant* or preterm or newborn* or NICU or NICUS or baby or babies or perinat* or PICU).ti,ab.	2
	**#1 or #2**	3
**Thrombosis**		
	thrombosis/ or exp venous thrombosis/ or thromboembolism/ or venous thromboembolism/	4
	(thrombos* or thrombus or thrombi or phlebothrombo* or phlebo thrombo* or (blood adj3 clot*) or venothrombo* or thromboembol* or thrombo embol* or DVT or VTE).ti,ab.	5
	**#4 OR #5**	6
**Risk factors**		
Controlled vocabulary	exp risk/ or causality/ or precipitating factors/ or incidence/ or epidemiologic studies/ or exp cohort studies/	7
Free text	(risk* or factor* or incidence* or predict* or association* or correlat* or relation* or interrelation* or likely or likel#hood* or odds or probab* or determinant* or cause* or causal* or causation* or etiolog* or aetiol* or link* or trigger* or genesis or connect* or epidemiol* or retrospect* or prospect* or ((cohort* or concurrent) adj2 stud*) or follow-up or followup or longitudinal).ti,ab.	8
	**#7 or #8**	9
	**#3 and #6 and #9**	10
	**limit 10 to yr="1990 -Current"**	11
	congress.pt. or congresses as topic/ or letter.pt. or editorial.pt. or comment.pt. or "consensus development conference, NIH".pt. or "consensus development conferences, NIH as topic"/ or consensus development conferences as topic/ or "consensus development conference".pt. or case reports.pt.	12
	(congress* or letter or letters or editorial* or comment or comments or commentary or viewpoint or consensus development conference* or (case adj2 (report* or studies))).ti,ab.	13
	**#12 or #13**	14
	**#11 not #14**	15

b) EMBASE

Concepts	Research strategy keywords	# Research
**Neonates**		
Controlled vocabulary	exp infant/ or neonatal intensive care unit/	1
Free text	(neonate* or neonatal or infant* or preterm or newborn* or NICU or NICUS or baby or babies or perinat* or PICU).ti,ab.	2
	**#1 or #2**	3

© 2026 Pelland-Marcotte MC et al. *JAMA Network Open*.

**Thrombosis**		
	thrombosis/ or exp vein thrombosis/ or thromboembolism/ or venous thromboembolism/	4
	(thrombos* or thrombus or thrombi or phlebothrombo* or phlebo thrombo* or (blood adj3 clot) or venothrombo* or thromboembol* or thrombo embol* or DVT or VTE).ti,ab.	5
	**#4 OR #5**	6
**Risk factors**		
Controlled vocabulary	risk/ or statistical model/ or exp risk assessment/ or mortality risk/ or risk factor/ or causality/ or incidence/ or cohort analysis/ or follow up/ or longitudinal study/ or prospective study/ or retrospective study/	7
Free text	(risk* or factor* or incidence* or predict* or association* or correlat* or relation* or interrelation* or likely or likel#hood* or odds or probab* or determinant* or cause* or causal* or causation* or etiolog* or aetiol* or link* or trigger* or genesis or connect* or epidemiol* or retrospect* or prospect* or ((cohort* or concurrent) adj2 stud*) or follow-up or followup or longitudinal).ti,ab.	8
	**#7 or #8**	9
	**#3 and #6 and #9**	10
	**limit 10 to yr="1990 -Current"**	11
	exp organization/ or exp conference paper/ or consensus development/ or case reports/	12
	(congress* or letter or letters or editorial* or comment or comments or commentary or viewpoint or consensus development conference* or (case adj2 (report* or studies))).ti,ab.	13
	(abstract or abstract report or conference or conference abstract or conference paper or "conference review" or data or data paper or editorial or erratum or letter or note or patent or tombstone).pt.	14
	**#12 or #13 or #14**	15
	**#11 not #15**	16

C) CINAHL

Concepts	Research strategy keywords	# Research
**Neonates**		
Controlled vocabulary	MH(infant+ or Intensive Care Units, Pediatric+ or Neonatal Nursing+)	1
Free text	TI(neonate* or neonatal or infant* or preterm or newborn* or NICU or NICUS or baby or babies or perinat* or PICU) or AB(neonate* or neonatal or infant* or preterm or newborn* or NICU or NICUS or baby or babies or perinat* or PICU)	2
	**#1 or #2**	3
**Thrombosis**		
	MH(thrombosis or catheter-related thrombosis or venous thrombosis+ or thromboembolism or venous thromboembolism)	4
	TI(thrombos* or thrombus or thrombi or phlebothrombo* or phlebo thrombo* or (blood N3 clot*) or venothrombo* or thromboembol* or thrombo embol* or DVT or VTE) or AB(thrombos* or thrombus or thrombi or phlebothrombo*	5

	or phlebo thrombo* or (blood N3 clot*) or venothrombo* or thromboembol* or thrombo embol* or DVT or VTE)	
	**#4 OR #5**	6
**Risk factors**		
Controlled vocabulary	MH(Risk factors or causality or incidence or Epidemiological Research or prospective studies+ or Multiple Logistic Regression or risk assessment)	7
Free text	TI(risk* or factor* or incidence* or predict* or association* or correlat* or relation* or interrelation* or likely or likel#hood* or odds or probab* or determinant* or cause* or causal* or causation* or etiolog* or aetiol* or link* or trigger* or genesis or connect* or epidemiol* or retrospect* or prospect* or ((cohort* or concurrent) N2 stud*) or follow-up or followup or longitudinal) or AB(risk* or factor* or incidence* or predict* or association* or correlat* or relation* or interrelation* or likely or likel#hood* or odds or probab* or determinant* or cause* or causal* or causation* or etiolog* or aetiol* or link* or trigger* or genesis or connect* or epidemiol* or retrospect* or prospect* or ((cohort* or concurrent) N2 stud*) or follow-up or followup or longitudinal)	8
	**#7 or #8**	9
	**#3 and #6 and #9**	10
	**limit 10 to yr="1990 -Current"**	11
	MH(Congresses and Conferences or Edit and Review or consensus or case studies)	12
	TI(congress* or letter or letters or editorial* or comment or comments or commentary or viewpoint or consensus development conference* or (case N2 (report* or studies)) OR AB(congress* or letter or letters or editorial* or comment or comments or commentary or viewpoint or consensus development conference* or (case N2 (report* or studies))	13
	**#12 or #13**	14
	**#11 not #14**	15

D) CLINICALTRIALS.GOV

Concepts	Research strategy keywords	# Research
Basic search interface	Condition: (thrombosis OR thromboses OR thrombotic OR thrombus OR thrombi OR phlebothrombosis OR phlebothrombotic OR "phlebo thrombosis" OR "phlebo thrombotic" OR venothrombosis OR venothrombotic OR thromboembolism OR thromboembolisms OR thromboembolic OR "thrombo embolism" OR "thrombo embolic" OR "blood clot" OR "blood clots" OR DVT OR "deep vein thrombosis" OR VTE OR "venous thromboembolism")<br>Age: from 0 to 28 days old	1
Advanced search	thrombosis neonates	2
	#1 OR #2	

© 2026 Pelland-Marcotte MC et al. *JAMA Network Open*.

eTable 2. Characteristics of included studies

**Characteristics**	**N (%)<br>N=60**
Study design	
Case-control	15 (25)
Prospective cohort study	16 (27)
Retrospective cohort study	26 (43)
Randomized controlled trial	1 (2)
Other	2 (3)
Year of publication	
1990-1999	5 (8)
2000-2009	11 (18)
2010-2019	21 (35)
2020-current	23 (38)
Geographical area	
North America	30 (50)
Europe	15 (25)
Asia	11 (18)
Other	4 (7)
Underlying conditions/clinical setting	
Population-based	1 (2)
General NICU population	34 (57)
Cardiac disease	12 (20)
Congenital anomaly	3 (5)
ECMO or extracorporeal circulation	3 (5)
Prematurity/low birth weight	5 (8)
Term or near-term infants	2 (3)
Routine imaging for venous thrombosis performed?	
Yes	29 (48)
No	27 (45)
Variable	4 (7)

Abbreviations: NICU: Neonatal Intensive Care Unit; ECMO: extracorporeal membrane oxygenation.

© 2026 Pelland-Marcotte MC et al. *JAMA Network Open*.

eTable 3. Study-level summary of included studies

Reference	Country of origin	Study design	Recruitment years	Population (Sample size)	Thrombosis definition
**Abdelsaimi, 2024**	Egypt	PCS	2023-2024	Neonates without congenital anomaly, requiring NICU admission (n=886)	Clinically suspected thrombosis, confirmed by relevant imaging
**Aiyagari, 2012**	USA	RCS	2007 - 2008	Infants with single ventricle cardiac disease undergoing surgical intervention (n=89)	Composite of: 1) CVC failure requiring placement of a new CVC, 2) radiologic diagnosis by US or angiogram, or 3) clinical signs of vascular occlusion leading to CVC removal
**Alten, 2012**	USA	RCS	2009 - 2011	Neonates with cardiac disease who underwent femoral central venous line attempts (n=115)	Radiologic diagnosis by US secondary to clinical suspicion (abnormal perfusion, venous stasis, or swelling) or incidental finding on US.
**Amankwah, 2014**	USA	CC	2006 - 2013	NICU admission of critically ill neonates (n=115)	Based on ICD-9 discharge codes and validated by review of radiologic records.
**Aronis, 2002**	Greece	CC	Not reported	Full term infants with cerebral infarct tested for thrombophilia (n=106)	Central nervous system thrombosis documented with magnetic resonance imaging
**Badur, 2021**	Germany	CC	2006 - 2016	Neonates requiring NICU admission, with a CVC (n=50)	Based on ICD-10 discharge codes
**Bhat, 2018**	USA	CC	2010 - 2014	Neonates requiring NICU admission (n=235)	Based on ICD-9 discharge codes
**Bhat, 2022**	USA	CC	2010 - 2016	Neonates requiring NICU admission (n=5 110)	Based on ICD-9 discharge codes
**Bhatia, 2022**	Canada	RCS	2014 - 2018	Neonates requiring NICU admission (n=186)	Not reported
**Chittithavorn, 2017**	Thailand	RCS	2006 - 2016	Neonates weighed <3 kg with functional univentricular heart and	Based on ICD-10 discharge codes

© 2026 Pelland-Marcotte MC et al. *JAMA Network Open*.

				underwent modified Blalock–Taussig shunt (n=85)	
**Chojnacka, 2022**	Poland	CC	2013 - 2016	Neonates with CVC insertion (n=44)	Persistent echodense structure within the vein, observed in two dimensions by US
**Claessens, 2018**	Netherlands	RCS	2009 - 2016	All neonates with critical CHD requiring cardiac surgery who underwent pre- and postoperative brain magnetic resonance imaging (n=40)	Based on ICD-10 discharge codes
**Eason, 2020**	USA	RCS	2015 - 2016	Neonates requiring NICU admission (n=77)	Based on ICD-9 discharge codes
**Easterlin, 2022**	USA	RCS	2016 - 2019	Neonates requiring NICU admission and discharged before 6 months of age (n=201 033)	Based on ICD-9 discharge codes
**El-Naggar, 2020**	Canada	CC	2014 - 2016	Neonates requiring NICU admission (n=39 971)	Based on medical chart review
**Emani, 2013**	USA	RCS	2010 - 2012	Neonates undergoing cardiac surgery (n=512)	Not reported
**Farag, 2024**	Egypt	PCS	2020-2021	Neonates requiring NICU admission, with a CVC (n=134)	Echodense structure within the heart or in vessels around CVC, observed in two dimensions
**Haddad, 2014**	Canada	RCS	2003 - 2009	Neonates requiring NICU admission, with a CVC for more than 14 days (n=645)	Shunt thrombosis
**Harrar, 2022**	USA	RCS	2013 - 2019	Neonates who had CHD repair with cardiopulmonary bypass and postoperative brain magnetic resonance imaging (MRI) (n=184)	Persistent echodense structure within the vein, observed in two dimensions by US
**Haumont, 2008**	Belgium	RCS	2002 - 2005	Neonates requiring NICU admission (n=339)	CSVT confirmed on MRV, T1, and US
**Huerta, 2023**	USA	RCS	2010 - 2014	Neonates with heterotaxy receiving Ladd procedures for intestinal malrotation (n=4 797)	CVC-related thrombosis identified in the electronic medical record

© 2026 Pelland-Marcotte MC et al. *JAMA Network Open*.

**Hundsdoerfer, 2003**	Germany	CC	1999 - 2001	Neonates with malrotation (n=85 304)	Based on ICD-10 discharge codes
**Hwang, 2020**	Korea	PCS	2017 - 2018	Neonates requiring NICU admission (n=137)	Any thrombus in the central venous or arterial systems on the basis of clinical signs and/or as an incidental finding
**Kim, 2001**	Korea	PCS	1995 - 1995	Neonates requiring NICU admission (n=102)	Clinical thrombosis events and evidence of thrombosis by echocardiography or cardiac catheterization
**Lambert, 2019**	USA	RCS	2013 - 2015	Neonates requiring NICU admission, with a CVC (n=766)	Blood clot within the vascular system related to the presence of a CVC as identified by Doppler US
**Maruyama, 2012**	Japan	CC	2004 - 2009	Extremely low birthweight infants (n=47)	Not reported
**Mehta, 1992**	USA	PCS	1984 - 1987	Newborns who required catheter placement (n=42)	Not reported
**Murai, 2002**	USA	PCS	1989 - 1993	Neonates admitted to special-care nursery (n=60)	Deep vein thrombosis
**Narang, 2009**	USA	RCS	1998 - 2004	Very low birthweight infants requiring CVC (n=210)	Not reported
**Navaratnam, 2023**	USA	RCS	2014 - 2018	Neonates undergoing cardiac surgery (n=86)	Not reported
**Nemati, 2013**	Iran	RCS	Not reported	Newborn infants with umbilical catheters (n=256)	Echogenic intraluminal thrombus at gray-scale US and absence of flow on color Doppler US images
**Ouellette, 2020**	Canada	RCS	1992 - 2016	All hospitalized neonates <=28 days born in Ontario (n=3 101 610)	Clinically identifiable renal thrombus
**Patregnani, 2018**	USA	RCS	2008 - 2014	Undergoing systemic-to pulmonary shunt placement. (n=75)	Venous thrombosis diagnosed by US
**Petaja, 1999**	Finland	RCS	1985 - 1998	Neonates undergoing cardiac surgery (n=242)	Thrombus along CVC detected by two-dimensional echocardiography
**Perez-Perez, 2023**	Spain	RCS	2014 - 2019	Neonates undergoing cardiac surgery (n=149)	Not reported

© 2026 Pelland-Marcotte MC et al. *JAMA Network Open*.

**Poppe, 2025**	Germany	RCS	2001-2022	Neonates with congenital heart disease undergoing Norwood surgery (n=360)	Any localized echogenic mass within the heart or in an extracardiac location (excluding CVC-related and ECMO-related thrombi)
**Puetz, 2009**	USA	RCS	Not reported	Critically ill neonates treated with either recombinant factor VIIa or fresh frozen plasma (n=234)	Thrombus detected by echocardiography
**Raets, 2013**	Netherlands	PCS	2010 - 2012	Preterm infants with gestational age of less than 29 wks admitted to the NICU (n=249)	Any arterial or venous thrombosis, pulmonary embolism, cerebral embolism, intracardiac thrombosis, shunt thrombosis or ECMO circuit thrombosis confirmed with imaging
**Rohr, 2014**	Germany	PCS	2012 - 2012	Low birthweight infants <1500g (n=72)	Not reported
**Rubio Longo, 2021**	Argentina	PCS	2015 - 2016	Neonates who required insertion of an CVCs (n=172)	Based on ICD-10 discharge codes (Renal vein thrombosis)
**Sakha, 2007**	Iran	PCS	2003 - 2004	Neonates with hyperbilirubinemia admitted and undergoing umbilical vein catheterization for exchange transfusion (n=50)	Not reported
**Salonvaara, 1999**	Finland	PCS	1990 - 1995	Neonates who required insertion of an indwelling CVC (n=44)	Diagnosed either: 1) phlebography (if symptoms of venous stasis were suspected); 2) right atrium assessment in postoperative echocardiography
**Schmidt, 1995**	Canada	RCS	1990 - 1993	Neonates diagnosed with venous or arterial large-vessel thrombosis (n=97)	Radiologically confirmed venous or arterial large-vessel thrombosis
**Schwartz, 1997**	USA	PCS	1993 - 1995	Tertiary care neonatal referral centers (n=173)	Not reported
**Shah, 2007**	Canada	RCS	2002 - 2005	Neonates requiring NICU admissions (n=201)	Thrombus detected by US and Doppler examination and/or by detecting absence of color Doppler signal or gray-scale thrombus visualization

© 2026 Pelland-Marcotte MC et al. *JAMA Network Open*.

**Sirachainan, 2017**	Thailand	RCS	1998 - 2015	Neonates requiring percutaneous CVC access (n=2 463)	Thrombus detected by repeat US examination in at least two planes
**Sobczak, 2021**	Poland	PCS	2016 - 2019	Neonates diagnosed with thrombosis and controls (n=129)	Presence of the following US criteria: echogenic material inside the vessel, lack of color flow in the Doppler venous mapping, lack of vein compressibility, increased vein diameter, and/or presence of collateral circulation.
**Sorg, 2021**	Germany	CC	2015 - 2017	Neonates requiring NICU admissions with an umbilical vein catheter (n=51)	Presence of echogenic intramural thrombosis
**Stein, 2019**	USA	RCS	2015 - 2016	Infants born at term and prematurely (n=124)	Cerebral sinovenous thrombosis (clinical and subclinical cases)
**Stewart, 2022**	USA	RCS	2005 - 2020	Neonates undergoing cardiac surgery (n=54)	Not reported
**Tan, 2011**	Canada	CC	1992 - 2006	Neonates with congenital heart disease cannulated onto ECMO (n=145)	Not reported
**Thornburg, 2007**	USA	RCS	2002 - 2005	Neonates (n=882)	Detection of a thrombus along the catheter path
**Tuckuviene, 2012**	Denmark	CC	1994 - 2006	Neonates requiring NICU admission (n=1 112)	Not reported
**Turebylu, 2007**	USA	PCS	2002 - 2003	Neonates with TE diagnosis (n=53)	Not reported
**Ulloa-Ricardez, 2015**	Mexico	CC	2008 - 2013	Neonates requiring NICU admission, undergoing umbilical catheter insertion (n=86)	Not reported
**Unal, 2012**	Turkey	CC	2010 - 2010	Neonates requiring NICU admission (n=46)	Thrombosis in right atrium and/or subclavian vein after CVC placement
**Uslu, 2010**	Turkey	RCT	2007 - 2008	Neonates requiring NICU admission (n=246)	Not reported
**White, 2020**	USA	RCS	2005 - 2009	Neonates requiring NICU admission, who needed CVC placement (n=549)	Not reported
**Xiong, 2025**	China	PCS	2022-2024	Neonates undergoing surgery for necrotizing enterocolitis, megacolon,	Occlusive or non-occlusive thrombus detected in at least one vessel,

© 2026 Pelland-Marcotte MC et al. *JAMA Network Open*.

				esophatresia atresia or atrial septal defect (n=188)	identified by Doppler US (both symptomatic or asymptomatic)
**Zhu, 2022**	China	CC	2014 - 2021	Infants with single ventricle physiology cardiac disease (n=63)	Thrombus leading to removal of peripherally inserted central catheter

Abbreviations: CVC: central venous catheter; CC: case-control study; ECMO: extracorporeal membrane oxygenation; PCS: prospective cohort study; RCS: retrospective cohort study; RCT: Randomized controlled trial; US: ultrasonography; NICU: Neonatal Intensive Care Unit.

© 2026 Pelland-Marcotte MC et al. *JAMA Network Open*.

eTable 4. Risk of bias in included studies

	Reference	Free of confounding	Free of selection bias	Adequate classification of exposure	No deviation from intended procedures	Adequate outcome data	Free of bias in outcome measurement	Free of selective reporting
1	Abdelsamei, 2024[1]	No	Yes	Yes	Yes	Yes	Yes	Yes
2	Aiyagari, 2012[2]	No	Yes	Yes	Yes	Yes	Yes	Yes
3	Alten, 2012[3]	No	Yes	Yes	Yes	Yes	Yes	Yes
4	Amankwah, 2014[4]	Yes	Yes	Yes	Yes	Yes	No	Yes
5	Aronis, 2002[5]	Yes	Yes	Yes	No	Yes	Yes	Yes
6	Badur, 2021[6]	No	Yes	Yes	Yes	Yes	Yes	Yes
7	Bhat, 2018[7]	No	Yes	Yes	Yes	Yes	No	Yes
8	Bhat, 2022[8]	No	Yes	Yes	Yes	Yes	No	Yes
9	Bhatia, 2022[9]	No	No	Yes	Yes	Yes	No	No
10	Chittihavorn, 2017[10]	No	Yes	Yes	Yes	Yes	No	Unclear
11	Chojnacka, 2022[11]	No	Yes	Unclear	Yes	Yes	No	Yes
12	Claessens, 2018[12]	No	Unclear	Yes	Yes	Yes	No	Yes
13	Eason, 2020[13]	Unclear	Yes	Unclear	Yes	Yes	Yes	Yes
14	Easterlin, 2022[14]	Yes	Yes	No	Yes	Yes	No	Yes
15	El-Naggar, 2020[15]	Yes	Unclear	Yes	Yes	Yes	Unclear	No
16	Emani, 2013[16]	No	Unclear	Yes	Yes	Unclear	Yes	Unclear
17	Farag, 2024[17]	No	Yes	Yes	Yes	Yes	Yes	No
18	Haddad, 2014[18]	No	Yes	Yes	Unclear	Yes	Yes	Yes
19	Harrar, 2022[19]	No	Yes	Yes	Yes	Yes	Yes	Yes
20	Haumont, 2008[20]	Yes	Yes	Yes	Yes	Yes	Yes	Yes
21	Huerta, 2023[21]	No	Yes	Yes	Yes	Unclear	Unclear	Yes
22	Hundsdoerfer, 2003[22]	No	No	Yes	Yes	Yes	No	Yes
23	Hwang, 2020[23]	Yes	Yes	Yes	Yes	Yes	No	Yes
24	Kim, 2001[24]	Yes	No	Yes	Yes	Yes	No	Yes
25	Lambert, 2019[25]	No	Yes	Yes	Yes	Yes	No	Yes

© 2026 Pelland-Marcotte MC et al. *JAMA Network Open*.

	Reference	Free of confounding	Free of selection bias	Adequate classification of exposure	No deviation from intended procedures	Adequate outcome data	Free of bias in outcome measurement	Free of selective reporting
26	Maruyama, 2012[26]	No	Yes	Yes	Yes	Yes	Yes	No
27	Mehta, 1992[27]	No	Yes	Unclear	Yes	Yes	Yes	Yes
28	Murai, 2002[28]	Yes	Yes	Yes	Yes	Yes	Yes	Yes
29	Narang, 2009[29]	No	Yes	Yes	Yes	Yes	Yes	No
30	Navaratnam, 2023[30]	No	Yes	Yes	Yes	Yes	No	Yes
31	Nemati, 2013[31]	No	Yes	Yes	Yes	Yes	Yes	Yes
32	Ouellette, 2020[32]	No	Yes	Yes	Yes	Yes	Unclear	Yes
33	Patregnani, 2018[33]	No	Yes	Yes	Yes	Yes	No	Yes
34	Petaja, 1999[34]	Yes	No	Yes	Unclear	Yes	Unclear	Yes
35	Perez-Perez, 2023[35]	No	Unclear	Unclear	Yes	Yes	Yes	Yes
36	Poppe, 2025[36]	No	Yes	Yes	Yes	Yes	Unclear	Unclear
37	Puetz, 2009[37]	No	No	Yes	Unclear	Yes	No	Yes
38	Raets, 2013[38]	No	Yes	Yes	Yes	Yes	Yes	No
39	Rohr, 2014[39]	No	Yes	Yes	Unclear	Yes	Yes	Yes
40	Rubio Longo, 2021[40]	No	Yes	Yes	Yes	Yes	Yes	No
41	Sakha, 2007[41]	No	No	Yes	Yes	Yes	Unclear	No
42	Salonvaara, 1999[42]	No	No	Yes	Yes	Yes	Yes	Yes
43	Schmidt, 1995[43]	Yes	Yes	Yes	Yes	No	Yes	Yes
44	Schwartz, 1997[44]	Yes	Yes	Yes	Yes	Unclear	Yes	Yes
45	Shah, 2007[45]	Yes	Yes	Yes	Yes	Unclear	Yes	Yes
46	Sirachainan, 2018 [46]	No	Yes	Yes	Yes	Yes	Yes	Yes
47	Sobczak, 2021[47]	No	Yes	Yes	Yes	Yes	Yes	Yes
48	Sorg, 2021[48]	No	Unclear	Yes	Yes	Yes	Yes	Yes
49	Stein, 2019[49]	Yes	Yes	Yes	Yes	Yes	Yes	Yes
50	Stewart, 2022[50]	Yes	Yes	Yes	Yes	Yes	Yes	Yes
51	Tan, 2011[51]	No	Yes	Yes	Yes	No	Yes	Yes

© 2026 Pelland-Marcotte MC et al. *JAMA Network Open*.

	Reference	Free of confounding	Free of selection bias	Adequate classification of exposure	No deviation from intended procedures	Adequate outcome data	Free of bias in outcome measurement	Free of selective reporting
52	Thornburg, 2007[52]	No	Yes	Yes	Yes	Yes	Yes	Yes
53	Tuckuviene, 2012[53]	No	Yes	No	Yes	Yes	Yes	Yes
54	Turebylu, 2007[54]	Yes	Yes	Yes	Yes	Yes	Yes	Yes
55	Ulloa-Ricardez, 2015[55]	No	No	Yes	Yes	Yes	No	Yes
56	Unal, 2012[56]	Yes	Yes	Yes	Yes	Yes	Yes	Yes
57	Uslu, 2010[57]	Yes	Yes	Yes	Yes	Yes	Yes	Yes
58	White, 2020[58]	Yes	Yes	Yes	Yes	Yes	No	Yes
59	Xiong, 2025[59]	Yes	Yes	Yes	Yes	Yes	Yes	Yes
60	Zhu, 2022[60]	No	Yes	Yes	Yes	Yes	Yes	No

© 2026 Pelland-Marcotte MC et al. *JAMA Network Open*.

eFigure 1: Flow chart of included studies

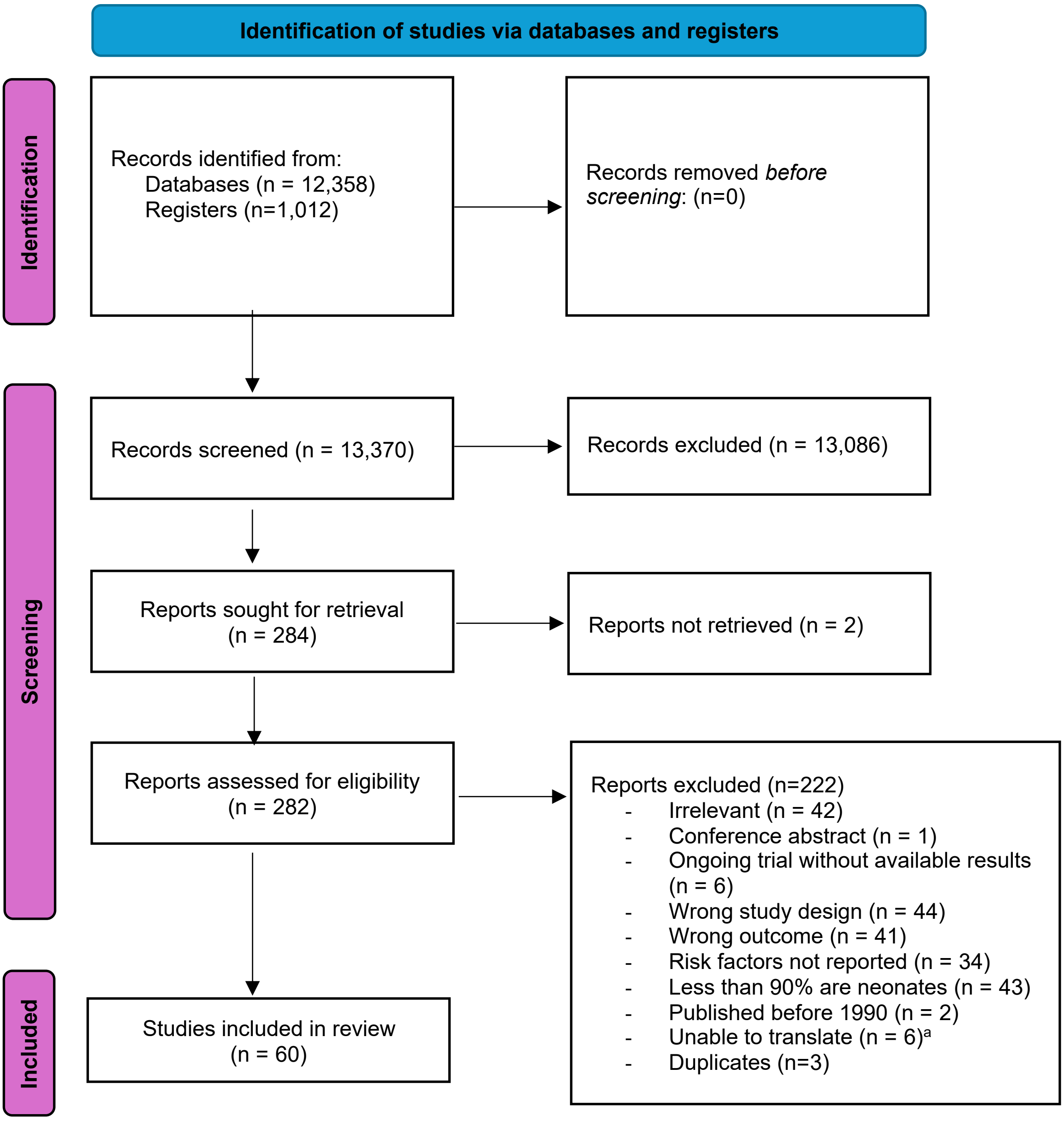

[a] Language unamenable to software-based translation.

© 2026 Pelland-Marcotte MC et al. *JAMA Network Open*.

eFigure 2. Forest plot of the effect of birthweight on the risk of venous thrombosis in neonates

a. With birthweight categorized as low vs. normal

Study	Sample size	Weight	log(Odds Ratio)
Haddad, 2014	645	56.85%	0.61 [0.10, 1.11]
Chittithavorn, 2017	85	9.05%	0.71 [-0.55, 1.97]
Ulloa-Ricardez, 2015	86	18.79%	0.87 [-0.01, 1.74]
Tuckuviene, 2012	384	15.32%	0.97 [-0.00, 1.94]
RE Model		100.00%	0.72 [0.34, 1.10]

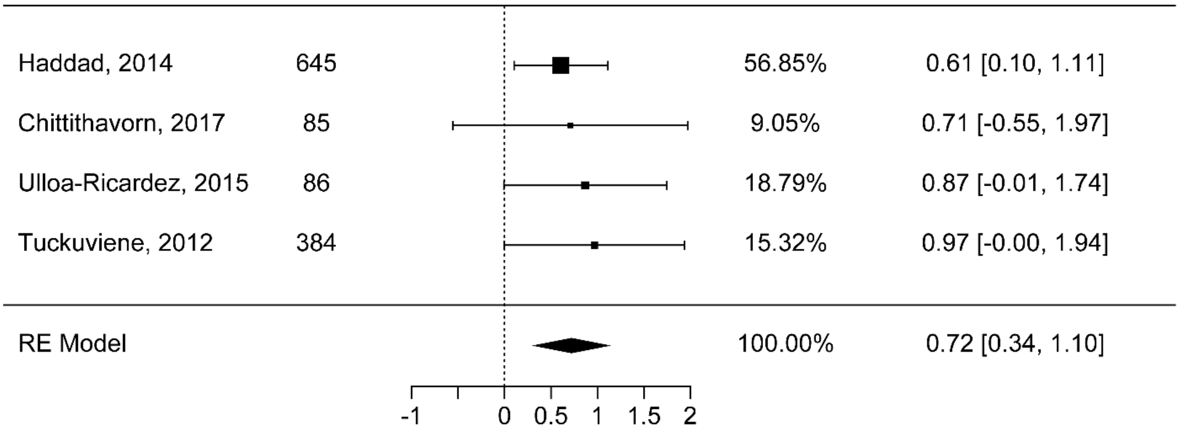

Back-transform log(OR) to OR: 2.054 (95% CI: 1.406-3.002)

b. With birthweight as a continuous variable, per g

Study	Simple size	Weight	Mean difference
Mehta, 1992	42	1.34%	-522.00 [-833.09, -210.91]
Shah, 2007	201	4.80%	-84.00 [-248.45, 80.45]
Raets, 2013	249	7.57%	-83.90 [-214.85, 47.05]
Ouellette, 2020	3 010 610	4.41%	-81.33 [-252.88, 90.22]
Narang, 2009	210	14.21%	-37.00 [-132.58, 58.58]
White, 2020	549	61.39%	-33.34 [-79.33,12.65]
Unal, 2012	46	4.09%	0.16 [-177.97, 178.29]
Eason, 2020	77	0.28%	27.00 [-649.97, 703.97]
Zhu, 2022	63	0.70%	110.84 [-318.37, 540.05]
Huang, 2020	137	0.91%	161.80 [-215.48, 539.08]
Harrar, 2022	178	0.28%	166.67 [-520.11, 853.45]
RE Model		100.00%	-43.91 [-79.94, -7.87]

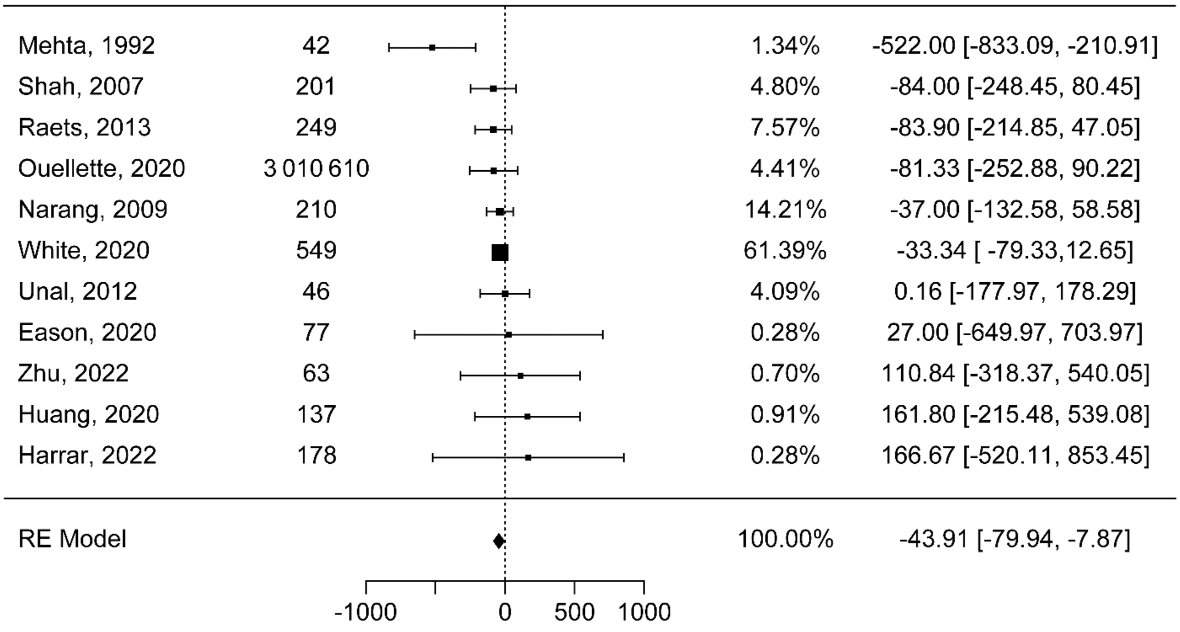

LEGEND : The analysis was carried out using the log odds ratio (S2a) and mean difference as the outcome measure (S2b). Amount of heterogeneity estimated using the restricted maximum likelihood estimator : Tau2=0.0 for both analyses.

© 2026 Pelland-Marcotte MC et al. *JAMA Network Open*.

eFigure 3. Forest plot of the effect of gestational age on the risk of venous thrombosis in neonates

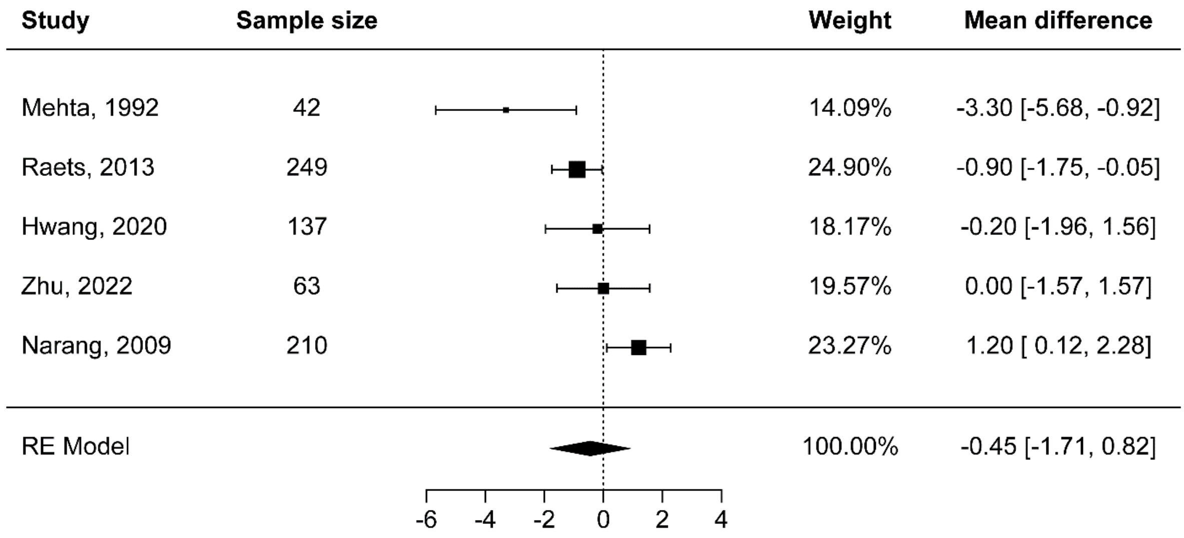

LEGEND: The analysis was carried out using the mean difference per week of gestation age as outcome measure. The amount of heterogeneity was estimated using the restricted maximum likelihood estimator: Tau^2=1.4882.

© 2026 Pelland-Marcotte MC et al. *JAMA Network Open*.

eFigure 4. Forest plot of the effect of sex (male vs. female) on the risk of venous thrombosis in neonates

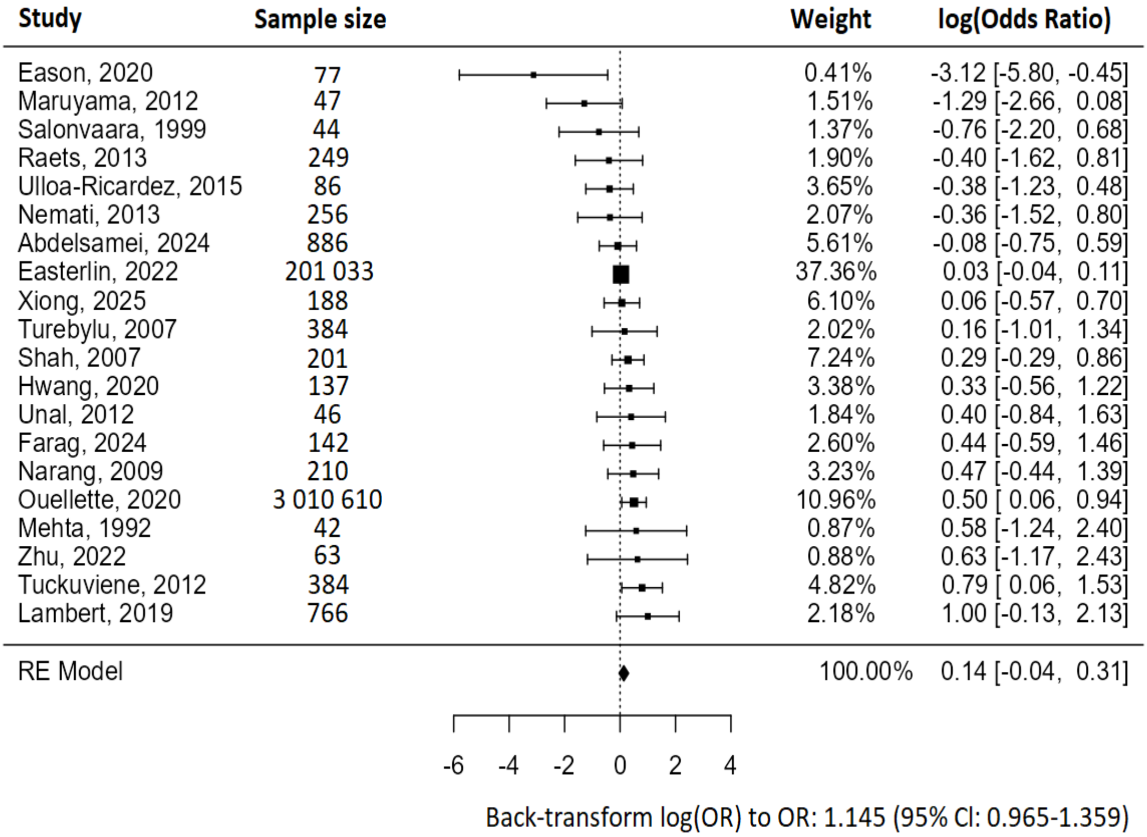

LEGEND: The analysis was carried out using the log odds ratio as outcome measure. The amount of heterogeneity was estimated using the restricted maximum likelihood estimator: Tau^2=0.0189.

© 2026 Pelland-Marcotte MC et al. *JAMA Network Open*.

eTable 5. Additional clinical factors associated with neonatal venous thrombosis

Reference	Study design	Population (Sample size)	Definition of VTE	Definition of exposure	Proportion of patients with factor in patients with VTE vs. Controls (%)[a]	OR (95% CI)[a]	p-value
	Chylothorax						
Perez-Perez, 2023[35]	RCS	Neonates undergoing cardiac surgery (n=149)	Not reported	Determined using pleural effusion values	24/31 vs. 27/118	**10.0 (3.6-28.0)**	**<0.001**
	Cardiac disease and related variables						
Chittithavorn, 2017[10]	PCS	Neonates undergoing mBTS (n=85)	Intra-cardiac shunt thrombosis	NR	8/12 vs. 11/73	**10.58 (1.86-60.22)**	**<0.01**
Chittithavorn, 2017[10]	PCS	Neonates undergoing mBTS (n=85)	Intra-cardiac shunt thrombosis	No heparin for >4 hours post-operatively	6/12 vs 3/73	**18.07 (2.48-131.86)**	**<0.01**
Eason, 2020[13]	PCS	Neonates with congenital heart disease requiring ECLS (n=77)	Catheter-related thrombosis, documented in medical record	Single vs. biventricular physiology	3/12 vs. 5/65	**10.29 (1.70-62.13)**	**0.01**
Ouellette, 2020[32]	RCS	Hospitalized neonates (n=3101610)	Clinically identifiable renal thrombus	Cardiac disease identified by ICD-9 and ICD-10 discharge codes	25/85 vs. 40372/3010525	**9.09 (5.05-16.4)**	**<0.001**
Poppe, 2025[36]	RCS	Neonates undergoing Norwood procedure (n=360)	Echogenic mass, intra-cardiac or in adjacent vessels	Restrictive atrial septal defect	24/42 vs. 98/318	**2.61 (1.33-5.12)**	**0.005**
White, 2020[58]	RCS	Neonates with single ventricle physiology conditions (n=549)	Any of: thromboembolism, superior vena cava occlusion, or inferior vena cava occlusion	ECLS bypass time, by 10-minute increments	NR	**1.07 (1.01-1.12)**	**0.022**

© 2026 Pelland-Marcotte MC et al. *JAMA Network Open*.

White, 2020[58]	RCS	Neonates with single ventricle physiology conditions (n=549)	Any of: thromboembolism, superior vena cava occlusion, or inferior vena cava occlusion	RVPAS, compared to MBTS	NR	1.06 (0.63-1.80)	0.82
Zhu, 2022[60]	CC	Prematurity (n=63)	PICC-related thrombosis, not further described	Cardiac insufficiency	1/7 vs. 1/56	**29.463 (NR)**	**0.035**
	Disease severity (global measure)						
Eason, 2020[13]	PCS	Neonates with congenital heart disease requiring ECLS (n=77)	Catheter-related thrombosis, documented in medical record	SNAPPE-II : score for neonatal acute physiology with perinatal extension- II	Med (IQR):<br>5 (5-28) vs 5 (0.23)	1.01 (1.00-1.10)	0.21
	Length of stay						
Abdelsamei, 2024[1]	PCS	Neonates admitted to NICU (n=886)	Clinically suspected thrombosis, confirmed by imaging	Duration of NICU admission, in days	NR	**1.11 (1.07-1.15)**	**0.001**
	Multiple births						
Tuckuviene, 2012[53]	CC	Neonates admitted to NICU (n=1112)	Clinically suspected thrombosis, confirmed by imaging, surgery or autopsy	Being a twin or a triplet	5/38 vs 3/346[b]	**7.1 (1.1-48.1)**	**0.045**
	Respiratory distress syndrome						
Bhatia, 2022[9]	RCS	Neonates admitted to NICU (n=186)	NR	NR	103/186 vs. NR	**2.41 (1.62-3.59)**	**<0.001**
Ouellette, 2020[32]	RCS	Hospitalized neonates (n=3101610)	Clinically identifiable renal thrombus	Identified by ICD-9 and ICD-10 discharge codes	44/85 vs. 204192/3010525	**8.01 (4.90-13.10)**	**<0.001**
	Twin-to-twin transfusion syndrome						
Zhu, 2022[60]	CC	Prematurity (n=63)	PICC-related thrombosis, not further described	Donor status	1/7 vs. 1/56	**29.463 (NR)**	**0.035**

© 2026 Pelland-Marcotte MC et al. *JAMA Network Open*.

Abbreviations: CC: case control; CI: confidence interval; CVC: central venous catheter; ECLS: extracorporeal life support; ICD: international classification of disease; mBTS: modified Black-TaussigtThomas shunt; NICU: neonatal intensive care unit; NR: not reported; PCS: prospective cohort study; OR: odds ratio; PICC: peripherally inserted central catheter; RCS: retrospective cohort study; RVPAS: right ventricle to pulmonary artery shunt; VTE: Venous thromboembolism.

[a] Statistically significant associations are emboldened.

[b] Data were not available for all cases and controls.

© 2026 Pelland-Marcotte MC et al. *JAMA Network Open*.

eFigure 5. Sensitivity analyses of the association between VTE and demographic variables – prospective studies only

a. Birthweight, in grams

Study	Sample size	Weight	Mean difference
Mehta, 1992	42	27.22%	-522.00 [-833.09, -210.91]
Raets, 2013	249	35.85%	-83.90 [-214.85, 47.05]
Eason, 2020	77	13.00%	27.00 [-649.97, 703.97]
Hwang, 2020	137	23.92%	161.80 [-215.48, 539.08]
RE Model		100.00%	-129.95 [-429.99, 170.10]

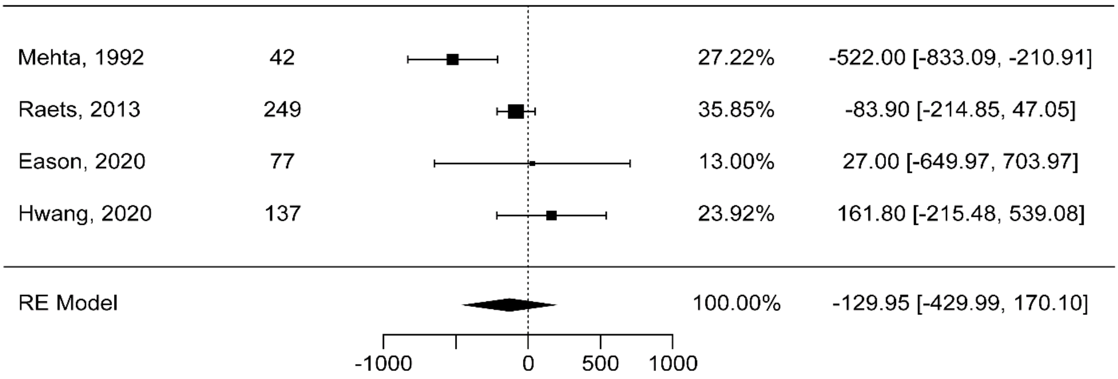

b. Gestational age, in weeks

Study	Sample size	Weight	Mean difference
Mehta, 1992	42	21.71%	-3.30 [-5.68, -0.92]
Raets, 2013	249	48.00%	-0.90 [-1.75, -0.05]
Hwang, 2020	137	30.29%	-0.20 [-1.96, 1.56]
RE Model		100.00%	-1.21 [-2.61, 0.19]

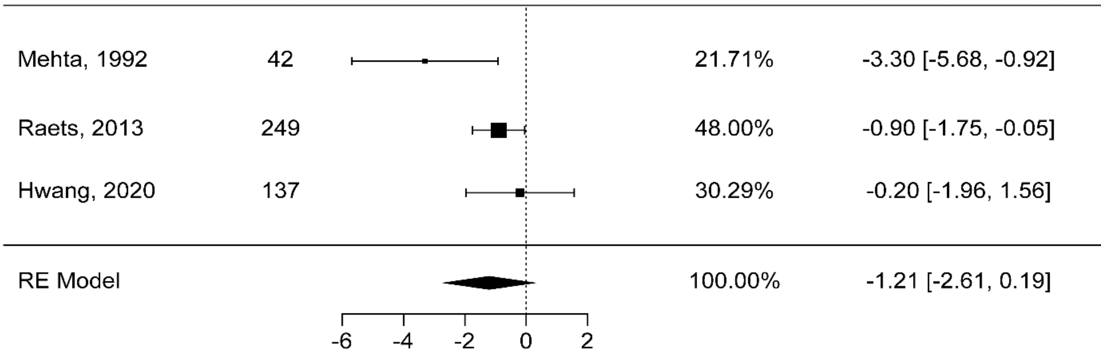

c. Sex, male vs. female

Study	Sample size	Weight	log(Odds Ratio)
Eason, 2020	77	1.52%	-3.12 [-5.80, -0.45]
Salonvaara, 1999	44	5.26%	-0.76 [-2.20, 0.68]
Raets, 2013	249	7.37%	-0.40 [-1.62, 0.81]
Abdelsamei, 2024	886	24.11%	-0.08 [-0.75, 0.59]
Xiong, 2025	188	26.62%	0.06 [-0.57, 0.70]
Turebylu, 2007	53	7.88%	0.16 [-1.01, 1.34]
Hwang, 2020	137	13.67%	0.33 [-0.56, 1.22]
Farag, 2024	142	10.29%	0.44 [-0.59, 1.46]
Mehta, 1992	42	3.28%	0.58 [-1.24, 2.40]
RE Model		100.00%	0.00 [-0.33, 0.33]

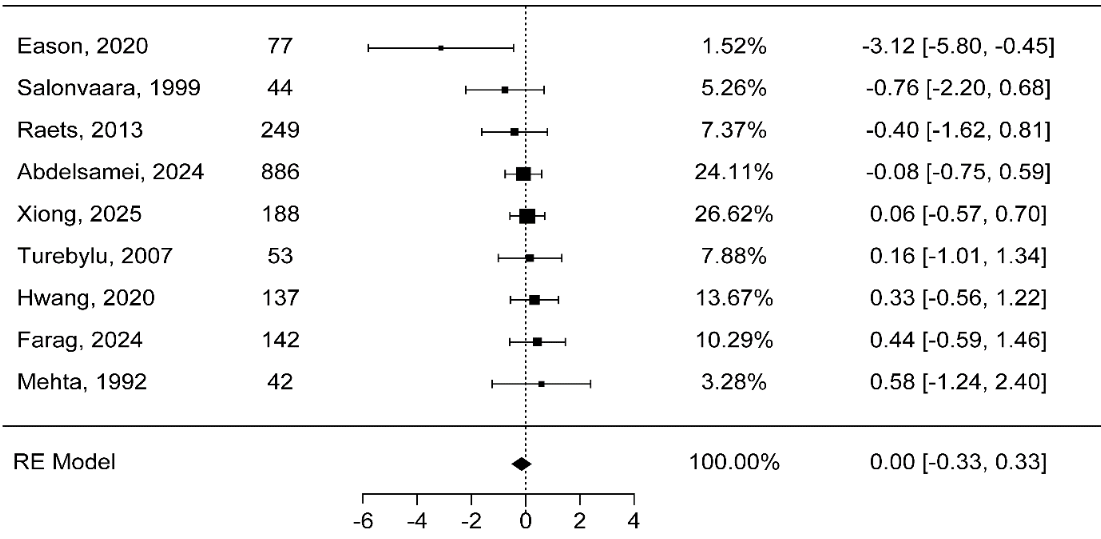

Back-transform log(OR) to OR: 1.002 (95% CI: 0.721-1.393)

LEGEND : The analyses were carried out using the mean difference (S5a, S5b) or log odds ratio (S5c) as outcome measures. Amount of heterogeneity estimated using the restricted maximum likelihood estimator : a) Tau2=60904.81; b) Tau2=0.8742, and c) Tau2=0.0.

© 2026 Pelland-Marcotte MC et al. *JAMA Network Open*.

eREFERENCES

1. Abdelsamei EM, Hakeem GLA, El Amin NM, Yousef MA, Ghalioub HS, Mohamed ZH. Assessment of the clinical and laboratory risk factors for thrombosis in neonates admitted to neonatal intensive care unit (two Egyptian tertiary centers experience). Multicenter Study. *Ann Hematol*. Nov 2024;103(11):4749-4757. doi:https://dx.doi.org/10.1007/s00277-024-06002-5
2. Aiyagari R, Song JY, Donohue JE, Yu S, Gaies MG. Central venous catheter-associated complications in infants with single ventricle: comparison of umbilical and femoral venous access routes. *Pediatric Critical Care Medicine*. 2012;13(5):549-553. doi:https://dx.doi.org/10.1097/PCC.0b013e31824fbdb4
3. Alten JA, Borasino S, Gurley WQ, Law MA, Toms R, Dabal RJ. Ultrasound-guided femoral vein catheterization in neonates with cardiac disease. *Pediatric Critical Care Medicine*. 2012;13(6):654-659. doi:https://dx.doi.org/10.1097/PCC.0b013e318250af0c
4. Amankwah EK, Atchison CM, Arlikar S, et al. Risk factors for hospital-sssociated venous thromboembolism in the neonatal intensive care unit. *Thrombosis Research*. 2014;134(2):305-309. doi:https://dx.doi.org/10.1016/j.thromres.2014.05.036
5. Aronis S, Bouza H, Pergantou H, Kapsimalis Z, Platokouki H, Xanthou M. Prothrombotic factors in neonates with cerebral thrombosis and intraventricular hemorrhage. *Acta Paediatrica Supplement*. 2002;91(438):87-91.
6. Badur CA, Buhrer C, Dame C. Adult Donor Blood Products as Risk Factors for Central Venous Catheter-associated Thromboembolism in Neonates: A Retrospective Case-Control Study. *Journal of Pediatric Hematology/Oncology*. 2021;43(2):e255-e259. doi:https://dx.doi.org/10.1097/MPH.0000000000001821
7. Bhat R, Kumar R, Kwon S, Murthy K, Liem RI. Risk Factors for Neonatal Venous and Arterial Thromboembolism in the Neonatal Intensive Care Unit-A Case Control Study. *Journal of Pediatrics*. 2018;195:28-32. doi:https://dx.doi.org/10.1016/j.jpeds.2017.12.015
8. Bhat R, Kwon S, Zaniletti I, Murthy K, Liem RI. Risk factors associated with venous and arterial neonatal thrombosis in the intensive care unit: a multicentre case-control study. *The Lancet Haematology*. 2022;9(3):e200-e207. doi:https://dx.doi.org/10.1016/S2352-3026(21)00399-9
9. Bhatia K, Solanki S, Paes B, Chan AKC, Bhatt MD. Risk factors for neonatal thrombosis: A retrospective study conducted in a single Canadian intensive care unit. *Pediatric Blood & Cancer*. 2022;69(6):e29668. doi:https://dx.doi.org/10.1002/pbc.29668
10. Chittithavorn V, Duangpakdee P, Rergkliang C, Pruekprasert N. Risk factors for in-hospital shunt thrombosis and mortality in patients weighing less than 3 kg with functionally univentricular heart undergoing a modified Blalock-Taussig shunt. *Interactive Cardiovascular & Thoracic Surgery*. 2017;25(3):407-413. doi:https://dx.doi.org/10.1093/icvts/ivx147
11. Chojnacka K, Krasinski Z, Wroblewska-Seniuk K, Mazela J. Catheter-related venous thrombosis in NICU: A case-control retrospective study. *Journal of Vascular Access*. 2022;23(1):88-93. doi:https://dx.doi.org/10.1177/1129729820983203
12. Claessens NHP, Algra SO, Jansen NJG, et al. Clinical and neuroimaging characteristics of cerebral sinovenous thrombosis in neonates undergoing cardiac surgery. *Journal of Thoracic & Cardiovascular Surgery*. 2018;155(3):1150-1158. doi:https://dx.doi.org/10.1016/j.jtcvs.2017.10.083
13. Eason AJ, Crethers D, Ghosh S, Stansfield BK, Polimenakos AC. Central Vascular Thrombosis in Neonates with Congenital Heart Disease Awaiting Cardiac Intervention. *Pediatric Cardiology*. 2020;41(7):1340-1345. doi:https://dx.doi.org/10.1007/s00246-020-02383-2

© 2026 Pelland-Marcotte MC et al. *JAMA Network Open*.

14. Easterlin MC, Li Y, Yieh L, et al. Predictors of venous thromboembolism among infants in children's hospitals in the United States: a retrospective Pediatric Health Information Study. *Journal of Perinatology*. 2022;42(1):103-109. doi:https://dx.doi.org/10.1038/s41372-021-01232-1
15. El-Naggar W, Yoon EW, McMillan D, et al. Epidemiology of thrombosis in Canadian neonatal intensive care units. *Journal of Perinatology*. 2020;40(7):1083-1090. doi:https://dx.doi.org/10.1038/s41372-020-0678-1
16. Emani S, Zurakowski D, Baird CW, Pigula FA, Trenor III C, Emani SM. Hypercoagulability markers predict thrombosis in single ventricle neonates undergoing cardiac surgery. *The Annals of thoracic surgery*. 2013;96(2):651-656.
17. Farag MM, Ghazal H, Radwan MM, El-Sayed NS. Catheters linked thrombosis in neonates: a single center observational study. Observational Study. *Italian Journal of Pediatrics*. Aug 13 2024;50(1):147. doi:https://dx.doi.org/10.1186/s13052-024-01708-8
18. Haddad H, Lee KS, Higgins A, McMillan D, Price V, El-Naggar W. Routine surveillance ultrasound for the management of central venous catheters in neonates. *Journal of Pediatrics*. 2014;164(1):118-122. doi:https://dx.doi.org/10.1016/j.jpeds.2013.08.048
19. Harrar DB, Goss M, Donofrio MT, et al. Cerebral Sinus Venous Thrombosis in Infants after Surgery for Congenital Heart Disease. *Journal of Pediatrics*. 2022;248:59-65.e53. doi:https://dx.doi.org/10.1016/j.jpeds.2022.05.056
20. Haumont D, de Beauregard VG, Van Herreweghe I, Delanghe G, Ciardelli R, Haelterman E. A new technique for transumbilical insertion of central venous silicone catheters in newborn infants. *Acta Paediatrica*. 2008;97(7):988-990. doi:https://dx.doi.org/10.1111/j.1651-2227.2008.00786.x
21. Huerta CT, Saberi RA, Lynn R, et al. Outcomes After Ladd Procedures for Intestinal Malrotation in Newborns with Heterotaxy Syndrome. *Journal of pediatric surgery*. 2023;58(6):1095-1100. doi:https://dx.doi.org/10.1016/j.jpedsurg.2023.02.013
22. Hundsdoerfer P, Vetter B, Stover B, et al. Homozygous and double heterozygous Factor V Leiden and Factor II G20210A genotypes predispose infants to thromboembolism but are not associated with an increase of foetal loss. *Thrombosis and Haemostasis*. 2003;90(4):628-635. doi:https://dx.doi.org/10.1160/th03-02-0096
23. Hwang JH, Chung ML, Lim YJ. Incidence and risk factors of subclinical umbilical catheter-related thrombosis in neonates. *Thrombosis Research*. 2020;194:21-25. doi:https://dx.doi.org/10.1016/j.thromres.2020.05.034
24. Kim JH, Lee YS, Kim SH, Lee SK, Lim MK, Kim HS. Does umbilical vein catheterization lead to portal venous thrombosis? Prospective US evaluation in 100 neonates. *Radiology*. 2001;219(3):645-650.
25. Lambert I, Tarima S, Uhing M, Cohen SS. Risk Factors Linked to Central Catheter-Associated Thrombosis in Critically Ill Infants in the Neonatal Intensive Care Unit. *American Journal of Perinatology*. 2019;36(3):291-295. doi:https://dx.doi.org/10.1055/s-0038-1667377
26. Maruyama H, Kitajima H, Yonemoto N, Fujimura M. Frequent use of fresh frozen plasma is a risk factor for venous thrombosis in extremely low birth weight infants: a matched case-control study. *Acta Medica Okayama*. 2012;66(1):61-66.
27. Mehta S, Connors AF, Jr., Danish EH, Grisoni E. Incidence of thrombosis during central venous catheterization of newborns: a prospective study. *Journal of Pediatric Surgery*. 1992;27(1):18-22.
28. Murai DT. Are femoral Broviac catheters effective and safe? A prospective comparison of femoral and jugular venous broviac catheters in newborn infants. *Chest*. 2002;121(5):1527-1530.

© 2026 Pelland-Marcotte MC et al. *JAMA Network Open*.

29. Narang S, Roy J, Stevens TP, Butler-O'Hara M, Mullen CA, D'Angio CT. Risk factors for umbilical venous catheter-associated thrombosis in very low birth weight infants. *Pediatric Blood & Cancer*. 2009;52(1):75-79. doi:https://dx.doi.org/10.1002/pbc.21714
30. Navaratnam M, Mendoza JM, Zhang S, et al. Activated 4-Factor Prothrombin Complex Concentrate as a Hemostatic Adjunct for Neonatal Cardiac Surgery: A Propensity Score-Matched Cohort Study. *Anesthesia and analgesia*. 2023;136(3):473-482. doi:https://dx.doi.org/10.1213/ANE.0000000000006294
31. Nemati M, Gharehbaghi MM, Shakeri A, Nobari RT, Behravan N, Goldust M. Vein thrombosis associated with umbilical vascular catheters with color doppler. *Journal of Biological Sciences*. 2013;13(8):722-726. doi:https://dx.doi.org/10.3923/jbs.2013.722.726
32. Ouellette AC, Darling EK, Sivapathasundaram B, et al. Incidence, Risk Factors, and Outcomes of Neonatal Renal Vein Thrombosis in Ontario: Population-Based Cohort Study. *Kidney360*. 2020;1(7):640-647. doi:https://dx.doi.org/10.34067/KID.0000912019
33. Patregnani JT, Sochet AA, Zurakowski D, et al. Cardiopulmonary Bypass Reduces Early Thrombosis of Systemic-to-Pulmonary Artery Shunts. *World Journal for Pediatric & Congenital Heart Surgery*. 2018;9(3):276-282. doi:https://dx.doi.org/10.1177/2150135118755985
34. Petaja J, Peltola K, Rautiainen P. Disappearance of symptomatic venous thrombosis after neonatal cardiac operations during antithrombin III substitution. *Journal of Thoracic & Cardiovascular Surgery*. 1999;118(5):955-956; discussion 957.
35. Perez-Perez A, Vigil-Vazquez S, Gutierrez-Velez A, et al. Chylothorax in newborns after cardiac surgery: a rare complication? *European journal of pediatrics*. 2023;182(4):1569-1578. doi:https://dx.doi.org/10.1007/s00431-023-04808-5
36. Poppe A, Matsubara M, Palm J, et al. Thrombus formation after the Norwood procedure: Incidence, risk factors, and its impact on late outcomes. *Int J Cardiol Congenit Heart Dis*. Jun 2025;20:100575. doi:https://dx.doi.org/10.1016/j.ijcchd.2025.100575
37. Puetz J, Darling G, Brabec P, Blatny J, Mathew P. Thrombotic events in neonates receiving recombinant factor VIIa or fresh frozen plasma. *Pediatric Blood & Cancer*. 2009;53(6):1074-1078. doi:https://dx.doi.org/10.1002/pbc.22160
38. Raets MM, Sol JJ, Govaert P, et al. Serial cranial US for detection of cerebral sinovenous thrombosis in preterm infants. *Radiology*. 2013;269(3):879-886. doi:https://dx.doi.org/10.1148/radiol.13130401
39. Rohr SB, Sauer H, Gottschling S, et al. Non-neurological, steroid-related adverse events in very low birth weight infants: a prospective audit. *Swiss Medical Weekly*. 2014;144:w13954. doi:https://dx.doi.org/10.4414/smw.2014.13954
40. Rubio Longo MC, De Lucca PM, Goldsmit G, Farina D, Lipsich J, Rodriguez S. Catheter-related deep vein thrombosis in newborn infants. *Archivos Argentinos de Pediatria*. 2021;119(1):32-38. doi:https://dx.doi.org/10.5546/aap.2021.eng.32
41. Sakha SH, Rafeey M, Tarzamani MK. Portal venous thrombosis after umbilical vein catheterization. *Indian Journal of Gastroenterology*. 2007;26(6):283-284.
42. Salonvaara M, Riikonen P, Kekomaki R, Heinonen K. Clinically symptomatic central venous catheter-related deep venous thrombosis in newborns. *Acta Paediatrica*. 1999;88(6):642-646.
43. Schmidt B, Andrew M. Neonatal thrombosis: report of a prospective Canadian and international registry. *Pediatrics*. 1995;96(5 Pt 1):939-943.
44. Schwartz DS, Gettner PA, Konstantino MM, et al. Umbilical venous catheterization and the risk of portal vein thrombosis. *Journal of Pediatrics*. 1997;131(5):760-762.
45. Shah PS, Kalyn A, Satodia P, et al. A randomized, controlled trial of heparin versus placebo infusion to prolong the usability of peripherally placed percutaneous central venous

© 2026 Pelland-Marcotte MC et al. *JAMA Network Open*.

catheters (PCVCs) in neonates: the HIP (Heparin Infusion for PCVC) study. *Pediatrics*. 2007;119(1):e284-291.
46. Sirachainan N, Limrungsikul A, Chuansumrit A, et al. Incidences, risk factors and outcomes of neonatal thromboembolism. *Journal of Maternal-Fetal & Neonatal Medicine*. 2018;31(3):347-351. doi:https://dx.doi.org/10.1080/14767058.2017.1285892
47. Sobczak A, Dudzik A, Kruczek P, Kwinta P. Ultrasound Monitoring of Umbilical Catheters in the Neonatal Intensive Care Unit-A Prospective Observational Study. *Frontiers in Pediatrics*. 2021;9:665214. doi:https://dx.doi.org/10.3389/fped.2021.665214
48. Sorg AL, Von Kries R, Klemme M, et al. Incidence and risk factors of cerebral sinovenous thrombosis in infants. *Developmental Medicine & Child Neurology*. 2021;63(6):697-704. doi:https://dx.doi.org/10.1111/dmcn.14816
49. Stein ML, Quinonez LG, DiNardo JA, Brown ML. Complications of Transthoracic Intracardiac and Central Venous Lines in Neonates Undergoing Cardiac Surgery. *Pediatric Cardiology*. 2019;40(4):733-737. doi:https://dx.doi.org/10.1007/s00246-019-02057-8
50. Stewart LA, Klein-Cloud R, Gerall C, et al. Extracorporeal Membrane Oxygenation (ECMO) and its complications in newborns with congenital diaphragmatic hernia. *Journal of Pediatric Surgery*. 2022;57(8):1642-1648. doi:https://dx.doi.org/10.1016/j.jpedsurg.2021.12.028
51. Tan M, Deveber G, Shroff M, et al. Sagittal sinus compression is associated with neonatal cerebral sinovenous thrombosis. *Pediatrics*. 2011;128(2):e429-435. doi:https://dx.doi.org/10.1542/peds.2010-3896
52. Thornburg CD, Smith PB, Smithwick ML, Cotten CM, Benjamin DK, Jr. Association between thrombosis and bloodstream infection in neonates with peripherally inserted catheters. *Thrombosis Research*. 2008;122(6):782-785.
53. Tuckuviene R, Christensen AL, Helgested J, Hundborg HH, Kristensen SR, Johnsen SP. Infant, obstetrical and maternal characteristics associated with thromboembolism in infancy: a nationwide population-based case-control study. *Archives of Disease in Childhood Fetal & Neonatal Edition*. 2012;97(6):F417-422. doi:https://dx.doi.org/10.1136/archdischild-2011-300665
54. Turebylu R, Salis R, Erbe R, Martin D, Lakshminrusimha S, Ryan RM. Genetic prothrombotic mutations are common in neonates but are not associated with umbilical catheter-associated thrombosis. *Journal of Perinatology*. 2007;27(8):490-495.
55. Ulloa-Ricardez A, Romero-Espinoza L, Estrada-Loza Mde J, Gonzalez-Cabello HJ, Nunez-Enriquez JC. Risk Factors for Intracardiac Thrombosis in the Right Atrium and Superior Vena Cava in Critically Ill Neonates who Required the Installation of a Central Venous Catheter. *Pediatrics & Neonatology*. 2016;57(4):288-294. doi:https://dx.doi.org/10.1016/j.pedneo.2015.10.001
56. Unal S, Ekici F, Cetin, II, Bilgin L. Heparin infusion to prevent umbilical venous catheter related thrombosis in neonates. *Thrombosis Research*. 2012;130(5):725-728. doi:https://dx.doi.org/10.1016/j.thromres.2012.07.018
57. Uslu S, Ozdemir H, Comert S, Bolat F, Nuhoglu A. The effect of low-dose heparin on maintaining peripherally inserted percutaneous central venous catheters in neonates. *Journal of Perinatology*. 2010;30(12):794-799. doi:https://dx.doi.org/10.1038/jp.2010.46
58. White MH, Kelleman M, Sidonio RF, Jr., Kochilas L, Patel KN. Incidence and Timing of Thrombosis After the Norwood Procedure in the Single-Ventricle Reconstruction Trial. *Journal of the American Heart Association*. 2020;9(24):e015882. doi:https://dx.doi.org/10.1161/JAHA.120.015882
59. Xiong L, Tan Y, Yang X, et al. Catheter-related Internal Jugular Vein Thrombosis in Neonates and Long-term Consequences: A Prospective Cohort Study. *Anesthesiology*. Feb 01 2025;142(2):298-307. doi:https://dx.doi.org/10.1097/ALN.0000000000005250

© 2026 Pelland-Marcotte MC et al. *JAMA Network Open*.

60. Zhu W, Zhang H, Xing Y. Clinical Characteristics of Venous Thrombosis Associated with Peripherally Inserted Central Venous Catheter in Premature Infants. *Children*. 2022;9(8):28. doi:https://dx.doi.org/10.3390/children9081126

© 2026 Pelland-Marcotte MC et al. *JAMA Network Open*.
