# Supplementary material for: Factors Associated With Thromboembolism in Neonates: A Systematic Review and Meta-Analysis
Source: JAMA Netw Open. 2026 May 6;9(5):e2610908. doi: 10.1001/jamanetworkopen.2026.10908 (PMC13150647; doi:10.1001/jamanetworkopen.2026.10908)
Supplement: Supplement 2. — Data Sharing Statement [file jamanetwopen-e2610908-s002.pdf]

Data Sharing Statement

Pelland-Marcotte. Factors Associated With Thromboembolism in Neonates. *JAMA Netw Open*. Published May 06, 2026. doi:10.1001/jamanetworkopen.2026.10908

Data
Data available: Yes
Data types: Data (not involving human participants)
How to access data: Data will be shared upon reasonable request to the corresponding author.
When available: With publication

Supporting Documents
Document types: None

Additional Information
Who can access the data: Data will be shared upon reasonable request to the corresponding author.
Types of analyses: For any purpose
Mechanisms of data availability: With investigator support
